# Supplementary material for: High-Throughput Mechanistic Screening of Epigenetic Compounds for the Potential Treatment of Meningiomas
Source: J Clin Med. 2021 Jul 16;10(14):3150. doi: 10.3390/jcm10143150 (PMC8303324; doi:10.3390/jcm10143150)
Supplement: Supplementary file 1 [file jcm-10-03150-s001.zip › jcm-1250286 XML Supplementary.pdf]

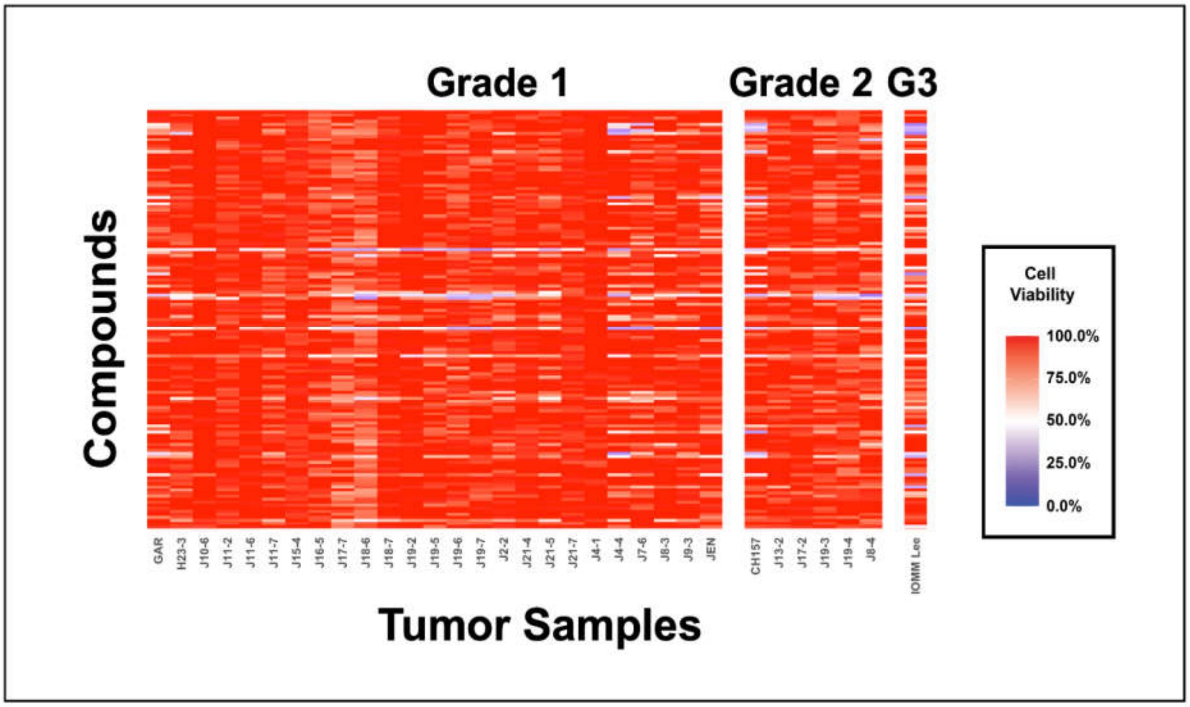

Supplementary Figure S1. Meningioma cohort sensitivity to epigenetic compounds separated by tumor grade. Heat map of meningiomas screened against the epigenetic compound panel, separated by grade 1, grade 2, and grade 3 (denoted as "G3") tumors.

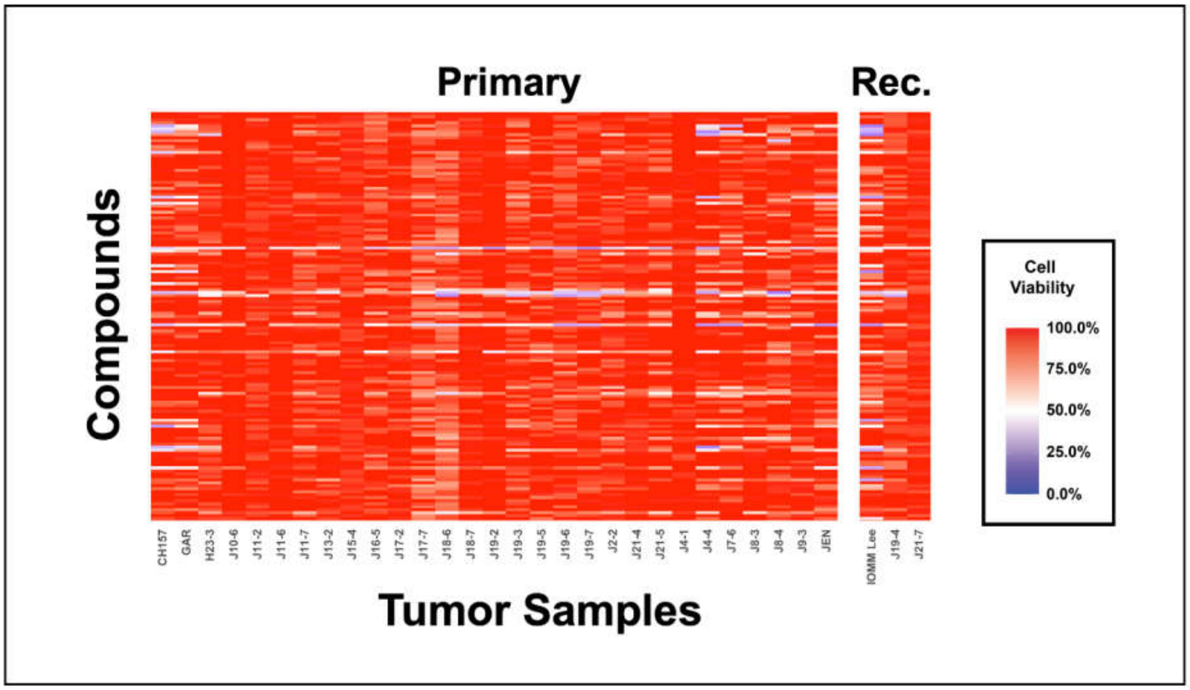

Supplementary Figure S2. Meningioma cohort sensitivity to epigenetic compounds separated by primary and recurrent tumors. Heat map of meningiomas screened against the epigenetic compound panel, separated by primary and recurrent (denoted as "Rec.") tumors.

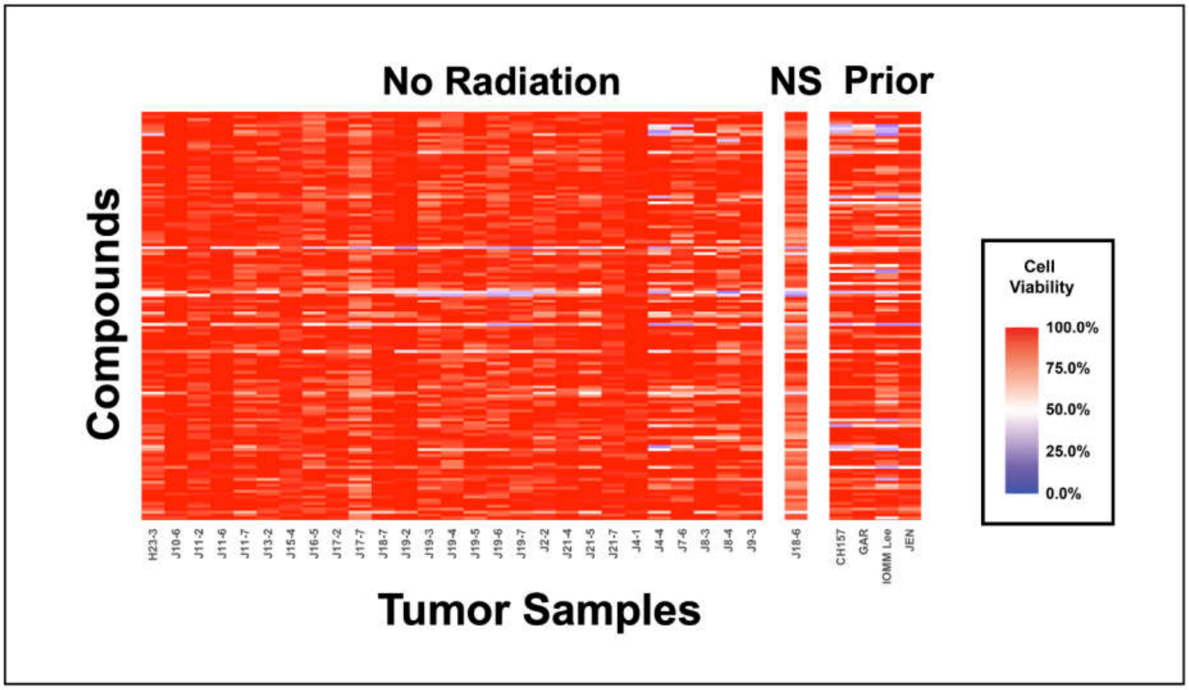

Supplementary Figure S3. Meningioma cohort sensitivity to epigenetic compounds separated by history of radiation. Heat map of meningiomas screened against the epigenetic compound panel, separated by tumors with no radiation, non-specified radiation (denoted as "NS"), and tumor with a prior history of radiation (denoted as "Prior").

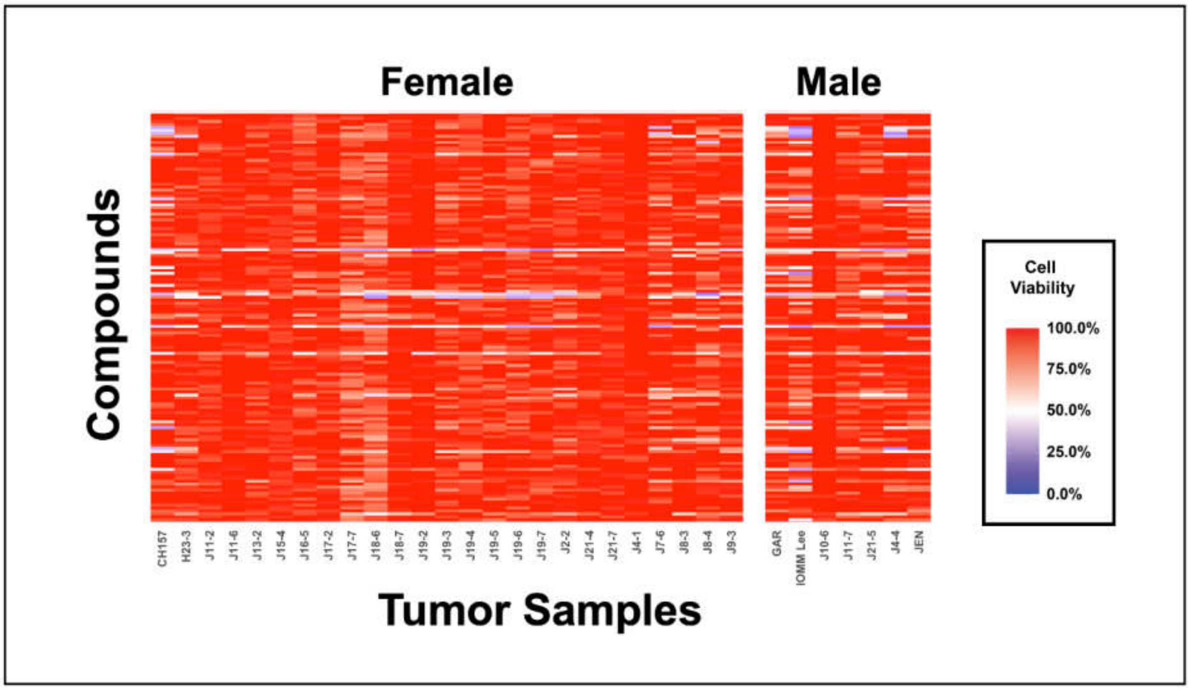

Supplementary Figure S4. Meningioma cohort sensitivity to epigenetic compounds separated by patient gender. Heat map of meningiomas screened against the epigenetic compound panel, separated by tumors with derived from female and male patients.

Supplementary Table S1. Cell viability for each epigenetic compound and meningioma sample. Complete table of the cell viability for each compound-sample pair. Cell viability is reported as a percentage normalized to the untreated tumor control and calculated from technical triplicate.

Compound	CH157	GAR	H23-3	IOMM Lee	J10-6	J11-2	J11-6	J11-7	J13-2	J15-4	J16-5	J17-2	J17-7	J18-6	J18-7
(-)-JQ1	97.0%	100.0%	100.0%	100.0%	100.0%	100.0%	98.8%	89.8%	96.1%	97.3%	100.0%	100.0%	82.9%	78.9%	96.9%
(-)-Neplanocin A	91.8%	96.4%	93.5%	47.7%	92.7%	100.0%	98.1%	100.0%	93.5%	100.0%	100.0%	96.3%	82.2%	92.7%	97.7%
(+)-Abscisic Acid	100.0%	100.0%	97.0%	100.0%	97.4%	94.0%	100.0%	87.3%	98.8%	95.3%	100.0%	100.0%	79.1%	91.0%	100.0%
(+)-JQ1	90.7%	100.0%	72.4%	95.3%	94.4%	90.6%	88.4%	66.9%	90.7%	100.0%	92.0%	97.2%	63.0%	48.3%	62.7%
(R)-PFI-2 (hydrochloride)	87.2%	79.4%	91.1%	88.6%	100.0%	97.6%	100.0%	100.0%	94.8%	95.3%	90.8%	93.6%	100.0%	85.1%	95.0%
1-Naphthoic Acid	100.0%	100.0%	96.2%	95.7%	100.0%	90.9%	99.1%	100.0%	100.0%	94.2%	100.0%	100.0%	83.7%	84.4%	99.7%
2-hexyl-4-Pentynoic Acid	92.0%	98.1%	90.9%	100.0%	100.0%	96.0%	100.0%	100.0%	100.0%	96.9%	90.1%	98.0%	93.6%	82.7%	100.0%
2-PCPA (hydrochloride)	99.4%	94.3%	100.0%	100.0%	100.0%	100.0%	99.1%	100.0%	96.7%	100.0%	100.0%	96.5%	100.0%	92.3%	100.0%
2,4-Pyridinedicarboxylic Acid	100.0%	100.0%	100.0%	98.0%	100.0%	100.0%	99.4%	96.0%	100.0%	93.8%	87.7%	100.0%	80.9%	76.7%	96.1%
2',3',5'-triacetyl-5-Azacytidine	99.3%	92.0%	100.0%	94.1%	94.9%	96.8%	97.6%	100.0%	91.5%	99.4%	100.0%	100.0%	90.2%	92.6%	100.0%
3-amino Benzamide	100.0%	100.0%	99.6%	100.0%	99.6%	100.0%	100.0%	83.6%	88.5%	93.9%	100.0%	96.4%	80.3%	92.2%	99.2%
3-Deazaneplanocin A	86.0%	79.6%	93.9%	70.8%	100.0%	92.4%	100.0%	100.0%	97.7%	95.7%	90.0%	96.1%	81.6%	79.8%	100.0%
3-Deazaneplanocin A (hydrochloride)	100.0%	92.9%	95.2%	63.8%	100.0%	100.0%	100.0%	100.0%	89.6%	97.3%	95.5%	82.0%	70.0%	75.4%	98.8%
3,3'-Diindolylmethane	90.1%	96.7%	76.4%	100.0%	100.0%	88.7%	99.4%	100.0%	100.0%	94.6%	95.5%	100.0%	100.0%	75.3%	95.5%
4-iodo-SAHA	88.6%	85.2%	99.3%	31.5%	100.0%	94.2%	100.0%	85.3%	69.8%	92.6%	100.0%	100.0%	70.7%	90.5%	100.0%
5-Azacytidine	100.0%	100.0%	91.3%	89.8%	100.0%	97.4%	96.3%	95.5%	99.7%	96.5%	100.0%	93.3%	88.5%	87.2%	100.0%
5-Methyl-2'-deoxycytidine	100.0%	93.5%	95.4%	95.9%	100.0%	96.0%	100.0%	100.0%	100.0%	94.3%	100.0%	100.0%	100.0%	85.6%	99.8%
5-Methylcytidine	94.8%	98.7%	91.6%	100.0%	100.0%	97.6%	100.0%	100.0%	100.0%	94.0%	100.0%	98.3%	100.0%	83.6%	100.0%
6-Thioguanine	47.4%	54.8%	89.4%	31.7%	83.2%	99.3%	96.8%	91.2%	98.0%	98.1%	79.5%	87.6%	78.4%	71.1%	89.5%
a-Hydroxyglutaric Acid (sodium salt)	100.0%	100.0%	97.8%	86.9%	100.0%	100.0%	97.5%	95.7%	100.0%	99.5%	89.5%	100.0%	99.4%	93.5%	97.7%
AGK2	100.0%	100.0%	94.1%	87.3%	100.0%	100.0%	100.0%	99.6%	100.0%	99.1%	89.3%	97.2%	85.8%	87.4%	96.5%
AGK7	100.0%	96.2%	92.0%	100.0%	100.0%	90.1%	100.0%	100.0%	100.0%	93.7%	97.8%	95.3%	100.0%	81.0%	100.0%
AK-7	98.2%	97.5%	100.0%	92.9%	100.0%	91.8%	100.0%	90.3%	100.0%	94.5%	77.1%	100.0%	100.0%	87.8%	100.0%
Anacardic Acid	100.0%	98.5%	93.1%	100.0%	100.0%	95.8%	100.0%	97.3%	99.4%	98.9%	95.7%	100.0%	87.7%	83.0%	95.9%
Apicidin	44.8%	70.9%	73.8%	32.1%	98.7%	100.0%	91.1%	80.2%	87.4%	92.2%	96.8%	94.4%	70.0%	60.2%	100.0%
BIX01294 (hydrochloride hydrate)	34.8%	43.7%	64.9%	43.4%	100.0%	100.0%	100.0%	68.1%	92.5%	97.3%	94.8%	100.0%	93.1%	94.3%	98.6%
BML-210	93.8%	90.3%	94.9%	100.0%	100.0%	97.5%	96.4%	100.0%	100.0%	94.5%	100.0%	100.0%	100.0%	85.6%	97.9%
BRD73954	100.0%	100.0%	96.1%	100.0%	100.0%	100.0%	95.7%	98.5%	97.5%	97.3%	100.0%	100.0%	100.0%	93.7%	98.3%
Bromosporine	99.3%	94.1%	80.9%	100.0%	100.0%	93.2%	100.0%	100.0%	100.0%	94.4%	100.0%	100.0%	100.0%	68.6%	90.5%
BSI-201	100.0%	100.0%	100.0%	100.0%	100.0%	93.6%	99.2%	95.0%	95.7%	95.1%	99.8%	100.0%	78.7%	73.0%	97.2%
Butyrolactone 3	100.0%	100.0%	89.9%	100.0%	100.0%	97.6%	100.0%	95.2%	99.8%	98.9%	90.5%	99.6%	87.8%	83.6%	96.0%
C646	100.0%	100.0%	91.0%	95.1%	98.6%	91.2%	99.9%	94.9%	96.9%	93.1%	90.4%	91.0%	100.0%	88.9%	100.0%
CAY10398	28.6%	56.1%	88.7%	50.1%	100.0%	92.2%	94.7%	81.2%	94.7%	94.5%	99.5%	93.3%	84.1%	83.3%	89.3%
CAY10591	90.6%	89.5%	98.5%	90.7%	100.0%	99.7%	100.0%	100.0%	100.0%	94.5%	100.0%	97.2%	100.0%	83.3%	98.7%
CAY10603	58.9%	56.2%	96.8%	30.2%	99.5%	90.0%	98.6%	86.2%	83.5%	93.3%	99.3%	84.8%	74.0%	93.9%	100.0%
CAY10669	100.0%	100.0%	100.0%	100.0%	98.0%	100.0%	100.0%	100.0%	100.0%	97.7%	97.9%	100.0%	86.8%	81.4%	100.0%
CAY10683	100.0%	100.0%	99.1%	100.0%	96.6%	96.6%	98.3%	100.0%	95.1%	96.3%	90.5%	100.0%	96.9%	96.3%	100.0%
CBHA	95.6%	96.2%	92.0%	86.3%	100.0%	97.6%	100.0%	88.8%	91.5%	99.3%	87.5%	90.5%	86.1%	83.9%	91.7%
CCG-100602	100.0%	100.0%	98.3%	94.5%	100.0%	100.0%	97.5%	93.4%	98.0%	98.1%	81.6%	96.0%	78.4%	85.4%	100.0%
Chidamide	89.5%	92.9%	98.1%	84.3%	100.0%	89.2%	100.0%	100.0%	100.0%	94.4%	94.4%	100.0%	100.0%	87.5%	100.0%
CI-994	87.3%	85.3%	100.0%	59.3%	100.0%	97.0%	98.5%	88.4%	85.9%	99.6%	97.0%	95.5%	94.5%	92.3%	100.0%
Cl-Amidine (hydrochloride)	100.0%	100.0%	100.0%	94.2%	100.0%	93.4%	100.0%	97.9%	100.0%	98.0%	100.0%	98.8%	90.5%	93.3%	100.0%
coumarin-SAHA	98.1%	98.6%	89.5%	73.3%	100.0%	90.7%	100.0%	89.7%	82.8%	98.7%	86.3%	95.9%	70.9%	89.4%	88.0%
CPI-203	99.3%	98.2%	55.6%	86.8%	78.2%	99.0%	93.5%	69.0%	95.3%	100.0%	70.1%	100.0%	73.9%	43.5%	66.2%
CPTH2 (hydrochloride)	100.0%	100.0%	100.0%	80.8%	100.0%	92.1%	100.0%	91.9%	93.2%	97.3%	94.0%	89.0%	79.8%	81.8%	95.6%
CUDC-101	86.1%	86.8%	85.8%	85.3%	100.0%	92.7%	100.0%	100.0%	93.7%	96.2%	100.0%	100.0%	100.0%	83.6%	93.4%
Daminozide	100.0%	100.0%	100.0%	100.0%	100.0%	89.4%	98.5%	94.8%	99.3%	95.8%	100.0%	92.6%	83.2%	99.0%	100.0%
Decitabine	99.5%	100.0%	94.8%	85.2%	100.0%	98.2%	100.0%	85.3%	87.9%	97.5%	83.5%	85.8%	82.8%	93.7%	95.7%
Delphinidin (chloride)	100.0%	100.0%	97.8%	93.4%	100.0%	100.0%	100.0%	100.0%	99.2%	100.0%	91.2%	98.7%	82.8%	90.1%	100.0%
DMOG	100.0%	90.2%	100.0%	77.9%	93.1%	100.0%	99.0%	100.0%	100.0%	98.3%	100.0%	100.0%	93.4%	84.7%	99.7%
Ellagic Acid	100.0%	100.0%	94.4%	97.5%	100.0%	93.6%	100.0%	89.0%	99.8%	97.8%	97.9%	83.1%	77.3%	95.5%	100.0%
EPZ005687	94.9%	97.9%	99.9%	94.9%	98.0%	94.0%	99.7%	100.0%	98.8%	100.0%	100.0%	100.0%	97.9%	98.8%	97.6%
EPZ5676	99.5%	100.0%	100.0%	99.8%	100.0%	100.0%	100.0%	100.0%	100.0%	92.3%	94.5%	100.0%	94.6%	100.0%	99.9%
Etoposide	100.0%	100.0%	93.6%	80.1%	99.7%	94.2%	99.8%	91.7%	100.0%	99.1%	97.5%	100.0%	97.3%	100.0%	100.0%
EX-527	86.2%	96.3%	96.4%	100.0%	100.0%	100.0%	100.0%	92.7%	100.0%	94.0%	99.1%	96.6%	98.4%	87.3%	98.9%
F-Amidine (trifluoroacetate salt)	100.0%	100.0%	100.0%	100.0%	100.0%	87.7%	100.0%	97.9%	98.2%	100.0%	100.0%	94.9%	87.1%	100.0%	100.0%
Garcinol	100.0%	100.0%	100.0%	87.9%	97.9%	92.9%	98.8%	100.0%	85.3%	98.5%	91.4%	91.1%	89.1%	100.0%	100.0%
Gemcitabine	45.6%	78.4%	86.4%	50.0%	82.4%	78.5%	86.3%	63.1%	66.6%	93.9%	50.0%	83.4%	62.1%	53.2%	100.0%
GSK-J1 (sodium salt)	100.0%	100.0%	100.0%	100.0%	100.0%	99.2%	95.2%	91.4%	100.0%	96.2%	100.0%	94.0%	88.0%	89.6%	95.6%
GSK-J2 (sodium salt)	94.5%	100.0%	100.0%	92.7%	100.0%	100.0%	98.9%	85.7%	93.1%	97.8%	100.0%	97.2%	89.3%	87.4%	97.2%
GSK-J4 (hydrochloride)	100.0%	100.0%	100.0%	100.0%	100.0%	100.0%	93.7%	100.0%	95.3%	100.0%	100.0%	96.3%	97.9%	95.1%	100.0%
GSK-LSD1 (hydrochloride)	98.5%	98.1%	98.7%	100.0%	100.0%	100.0%	99.5%	100.0%	100.0%	91.8%	100.0%	99.9%	100.0%	86.9%	98.9%
GSK126	100.0%	85.0%	99.0%	80.0%	98.7%	100.0%	99.2%	100.0%	100.0%	94.1%	92.1%	100.0%	95.9%	82.4%	97.9%
GSK2801	94.5%	93.9%	100.0%	99.5%	100.0%	91.1%	100.0%	100.0%	100.0%	94.4%	100.0%	100.0%	100.0%	90.4%	100.0%
GSK343	84.2%	97.0%	85.8%	100.0%	100.0%	96.7%	100.0%	97.6%	100.0%	92.3%	100.0%	100.0%	100.0%	84.1%	100.0%
GSK4112	100.0%	100.0%	100.0%	100.0%	100.0%	94.5%	97.7%	95.3%	92.3%	98.6%	100.0%	100.0%	82.3%	82.7%	98.3%
HC Toxin	37.7%	47.0%	51.1%	27.6%	61.9%	100.0%	61.6%	67.6%	71.6%	92.1%	50.1%	66.7%	41.7%	42.2%	64.3%
HNHA	98.5%	96.2%	100.0%	100.0%	94.5%	98.5%	97.2%	99.3%	100.0%	95.6%	94.3%	99.3%	83.8%	100.0%	100.0%
HPOB	97.2%	96.8%	100.0%	100.0%	100.0%	87.2%	100.0%	100.0%	100.0%	96.3%	91.8%	92.4%	100.0%	89.2%	97.7%
I-BET151	79.2%	91.2%	92.8%	100.0%	100.0%	92.7%	99.8%	92.8%	87.6%	100.0%	99.2%	91.7%	81.2%	85.3%	74.4%
I-BET762	84.1%	100.0%	68.2%	96.3%	100.0%	90.6%	94.2%	65.1%	89.5%	100.0%	68.3%	97.3%	71.3%	64.0%	78.2%
I-CBP112 (hydrochloride)	100.0%	98.3%	97.6%	74.7%	100.0%	100.0%	100.0%	97.6%	98.5%	98.6%	98.3%	100.0%	98.3%	99.0%	100.0%
IOX1	100.0%	100.0%	97.7%	88.7%	100.0%	94.2%	95.4%	90.0%	100.0%	98.2%	93.9%	92.3%	81.5%	83.9%	98.4%
Isoliquiritigenin	100.0%	100.0%	94.6%	100.0%	100.0%	97.6%	97.5%	100.0%	97.6%	100.0%	100.0%	100.0%	80.8%	75.7%	91.8%
ITF 2357	94.8%	98.1%	82.6%	54.7%	100.0%	95.2%	96.5%	91.3%	86.9%	100.0%	100.0%	99.9%	85.9%	90.8%	100.0%
JGB1741	100.0%	100.0%	100.0%	99.8%	100.0%	95.4%	100.0%	95.4%	98.3%	100.0%	100.0%	100.0%	90.4%	100.0%	100.0%
JIB-04	84.4%	90.5%	47.9%	72.7%	78.6%	53.0%	100.0%	98.6%	100.0%	82.2%	100.0%	93.0%	100.0%	25.7%	98.3%
LAQ824	35.3%	38.7%	52.0%	39.1%	61.4%	98.9%	78.0%	67.5%	46.0%	89.5%	72.7%	77.1%	48.5%	35.8%	60.4%
Lestaurtinib	96.5%	99.5%	79.6%	92.8%	99.2%	91.0%	87.1%	93.6%	73.5%	100.0%	70.0%	100.0%	75.6%	55.0%	66.2%
Lomeguatrib	100.0%	100.0%	100.0%	100.0%	100.0%	95.9%	96.9%	91.7%	96.1%	95.5%	100.0%	90.2%	76.1%	92.8%	96.0%
M 344	46.7%	66.0%	92.5%	56.8%	99.8%	99.4%	99.6%	88.6%	96.0%	93.7%	84.5%	88.6%	81.4%	92.6%	91.6%
MC 1568	100.0%	100.0%	99.3%	97.9%	99.6%	100.0%	97.0%	100.0%	100.0%	95.5%	100.0%	100.0%	92.0%	90.2%	97.9%
Methylstat (hydrate)	97.3%	92.0%	95.4%	79.8%	100.0%	88.8%	99.2%	85.7%	100.0%	92.8%	100.0%	100.0%	80.6%	82.3%	91.4%
MI-2 (hydrochloride)	100.0%	100.0%	100.0%	80.9%	98.5%	96.2%	99.8%	92.1%	99.9%	100.0%	75.5%	86.7%	100.0%	95.9%	98.4%
MI-nc (hydrochloride)	54.5%	43.9%	100.0%	24.2%	100.0%	95.9%	100.0%	85.4%	91.9%	93.9%	81.7%	87.3%	71.2%	100.0%	92.0%
Mirin	100.0%	91.0%	100.0%	91.0%	100.0%	100.0%	97.6%	94.2%	90.4%	96.5%	96.0%	100.0%	91.9%	93.0%	100.0%
MS-275	47.9%	75.9%	100.0%	50.3%	96.3%	100.0%	100.0%	84.7%	90.4%	90.8%	98.5%	100.0%	100.0%	99.7%	100.0%
N-Oxalylglycine	98.9%	97.0%	95.4%	100.0%	98.8%	95.8%	100.0%	100.0%	99.9%	100.0%	100.0%	100.0%	95.8%	89.1%	100.0%
Nicotinamide	100.0%	99.8%	99.8%	100.0%	100.0%	100.0%	99.4%	97.9%	99.8%	95.8%	99.2%	100.0%	83.4%	83.4%	97.9%
Octyl-a-ketoglutarate	100.0%	100.0%	100.0%	100.0%	100.0%	93.4%	98.1%	93.0%	92.5%	98.0%	100.0%	100.0%	77.9%	89.5%	100.0%
OTX015	92.1%	94.7%	74.9%	97.3%	100.0%	88.5%	100.0%	76.9%	100.0%	99.5%	87.7%	100.0%	93.2%	44.4%	81.1%
Oxamflatin	53.5%	65.4%	89.2%	57.9%	100.0%	97.3%	96.7%	89.6%	99.4%	92.0%	100.0%	93.8%	87.7%	88.2%	90.2%
Panobinostat	40.0%	55.0%	45.5%	46.6%	46.6%	94.8%	48.1%	53.9%	49.5%	89.4%	40.0%	59.1%	32.9%	31.8%	47.8%
PCI 34051	100.0%	100.0%	100.0%	100.0%	99.8%	98.1%	97.5%	99.7%	89.6%	95.4%	100.0%	100.0%	86.9%	89.8%	100.0%
PFI-1	100.0%	100.0%	85.0%	100.0%	100.0%	94.8%	96.6%	97.0%	95.4%	95.3%	99.6%	92.3%	76.3%	74.0%	99.9%
PFI-3	96.6%	94.1%	90.5%	100.0%	100.0%	95.4%	100.0%	100.0%	100.0%	94.3%	82.1%	100.0%	100.0%	84.2%	100.0%
Phthalazinone pyrazole	99.7%	88.7%	91.2%	75.2%	100.0%	100.0%	100.0%	97.8%	95.8%	100.0%	87.2%	100.0%	80.4%	73.0%	98.2%
Piceatannol	88.7%	97.2%	92.8%	97.4%	100.0%	100.0%	100.0%	96.7%	100.0%	94.0%	98.4%	97.9%	100.0%	79.8%	100.0%
Pimelic Diphenylamide 106	97.9%	84.2%	99.3%	75.0%	100.0%	93.9%	97.2%	98.8%	93.8%	95.2%	85.8%	100.0%	94.5%	81.1%	100.0%
Pyroxamide	91.5%	84.2%	98.7%	60.7%	95.9%	93.7%	100.0%	99.7%	94.2%	94.0%	100.0%	100.0%	92.8%	92.8%	100.0%
RG-108	98.1%	95.2%	100.0%	100.0%	93.2%	94.4%	96.6%	100.0%	100.0%	97.4%	96.6%	100.0%	98.2%	100.0%	100.0%
RGFP966	100.0%	97.5%	98.2%	100.0%	100.0%	97.3%	100.0%	95.3%	100.0%	94.0%	88.7%	100.0%	97.1%	86.8%	96.1%
RSC-133	88.1%	96.4%	100.0%	100.0%	100.0%	94.3%	100.0%	98.6%	100.0%	95.3%	98.9%	97.2%	100.0%	89.4%	95.3%
Rucaparib (phosphate)	100.0%	100.0%	93.2%	67.7%	100.0%	92.7%	96.8%	86.3%	91.2%	95.1%	100.0%	95.6%	78.1%	75.9%	98.4%
RVX-208	75.3%	95.4%	100.0%	98.3%	100.0%	92.1%	92.5%	96.8%	100.0%	98.0%	91.7%	95.8%	100.0%	96.3%	92.6%
S-(5'-Adenosyl)-L-methionine (tosylate)	100.0%	83.0%	97.3%	83.5%	100.0%	100.0%	99.0%	100.0%	99.2%	99.4%	89.0%	100.0%	96.6%	91.8%	98.7%
S-Adenosylhomocysteine	82.6%	89.9%	94.6%	80.7%	100.0%	94.5%	90.0%	100.0%	97.2%	97.0%	84.8%	100.0%	88.8%	100.0%	97.7%
SAHA	45.8%	51.6%	92.6%	53.3%	100.0%	93.7%	97.5%	93.9%	89.3%	90.9%	87.0%	100.0%	90.1%	87.4%	100.0%
Salermide	96.6%	98.2%	97.1%	98.5%	100.0%	99.3%	98.8%	96.3%	100.0%	96.9%	100.0%	96.8%	100.0%	88.8%	98.4%
SB939	38.0%	48.2%	85.7%	28.9%	95.0%	100.0%	95.2%	82.4%	88.5%	94.5%	86.4%	83.8%	65.7%	83.5%	100.0%
Scriptaid	92.3%	93.0%	97.9%	75.9%	99.8%	94.5%	100.0%	84.5%	91.1%	98.0%	85.2%	96.6%	84.8%	96.1%	92.1%
SGC-CBP30	100.0%	100.0%	94.7%	92.0%	100.0%	100.0%	99.0%	100.0%	99.3%	99.4%	95.0%	93.9%	92.7%	92.4%	98.4%
SGC0946	79.5%	96.9%	100.0%	97.3%	100.0%	89.2%	94.0%	100.0%	100.0%	99.9%	75.5%	97.0%	94.8%	100.0%	99.1%
SGI-1027	100.0%	82.9%	82.9%	78.5%	100.0%	99.4%	98.0%	100.0%	100.0%	100.0%	100.0%	99.2%	93.1%	93.1%	100.0%
Sinefungin	91.1%	86.8%	100.0%	92.0%	98.7%	90.5%	94.1%	100.0%	95.7%	93.5%	98.2%	100.0%	100.0%	97.0%	100.0%
SIRT1/2 Inhibitor IV	100.0%	100.0%	97.5%	99.7%	100.0%	94.0%	100.0%	100.0%	100.0%	96.4%	100.0%	100.0%	98.7%	88.5%	97.1%
Sirtinol	100.0%	96.9%	100.0%	100.0%	99.7%	88.8%	100.0%	96.2%	96.0%	93.8%	89.9%	100.0%	79.9%	91.2%	100.0%
Sodium 4-Phenylbutyrate	100.0%	100.0%	100.0%	81.8%	100.0%	99.0%	97.9%	100.0%	100.0%	93.6%	100.0%	91.7%	87.1%	78.7%	95.3%
Sodium Butyrate	98.2%	81.0%	98.6%	74.4%	98.1%	95.4%	100.0%	97.1%	89.4%	99.3%	100.0%	100.0%	86.0%	97.5%	100.0%
Splitomicin	100.0%	100.0%	89.8%	80.1%	100.0%	93.4%	100.0%	96.5%	93.8%	99.1%	90.3%	89.7%	80.4%	83.6%	96.5%
Suramin (sodium salt)	100.0%	100.0%	98.9%	100.0%	100.0%	100.0%	100.0%	100.0%	100.0%	95.5%	93.5%	97.8%	87.3%	85.5%	98.4%
Tenovin-1	100.0%	100.0%	98.6%	100.0%	100.0%	97.8%	100.0%	90.5%	96.6%	95.6%	88.7%	100.0%	88.4%	97.6%	95.1%
Tenovin-6	100.0%	100.0%	97.7%	99.6%	100.0%	95.8%	100.0%	89.4%	81.8%	98.1%	97.3%	100.0%	90.9%	77.7%	94.9%
trans-Resveratrol	93.4%	100.0%	98.4%	100.0%	100.0%	100.0%	97.9%	97.4%	100.0%	97.6%	100.0%	100.0%	100.0%	85.8%	99.7%
Trichostatin A	39.7%	62.7%	76.7%	51.1%	100.0%	100.0%	94.1%	88.2%	100.0%	97.8%	89.7%	100.0%	81.9%	79.4%	95.4%
Tubacin	85.4%	87.3%	100.0%	86.7%	100.0%	92.4%	100.0%	100.0%	100.0%	90.5%	92.5%	100.0%	97.3%	100.0%	100.0%
Tubastatin A	79.7%	95.3%	100.0%	100.0%	100.0%	84.1%	92.5%	100.0%	100.0%	98.4%	94.1%	97.1%	100.0%	99.6%	97.6%
Tubastatin A (trifluoroacetate salt)	98.1%	100.0%	100.0%	91.1%	99.2%	89.6%	97.8%	100.0%	88.6%	96.9%	99.4%	100.0%	90.0%	99.3%	100.0%
UNC0224	85.4%	81.9%	100.0%	55.9%	99.4%	96.4%	100.0%	100.0%	100.0%	94.1%	79.9%	100.0%	91.6%	100.0%	98.4%
UNC0321 (trifluoroacetate salt)	100.0%	100.0%	100.0%	94.1%	100.0%	100.0%	100.0%	100.0%	100.0%	97.4%	91.2%	97.1%	88.9%	88.1%	97.1%
UNC0631	78.7%	74.5%	40.4%	22.1%	100.0%	100.0%	100.0%	84.4%	86.1%	98.8%	90.5%	87.0%	75.4%	77.6%	94.5%
UNC0638	34.3%	78.2%	86.4%	31.9%	100.0%	98.8%	100.0%	74.0%	90.2%	96.1%	88.7%	96.8%	78.7%	80.9%	92.5%
UNC0642	43.8%	59.8%	90.3%	34.6%	100.0%	100.0%	99.7%	100.0%	98.6%	98.8%	84.2%	99.1%	99.4%	86.3%	100.0%
UNC0646	31.9%	53.0%	91.1%	22.4%	100.0%	96.4%	100.0%	82.2%	88.3%	90.6%	91.4%	100.0%	75.8%	89.2%	95.5%
UNC1215	85.4%	98.3%	97.5%	100.0%	100.0%	96.9%	100.0%	100.0%	96.1%	93.4%	79.1%	100.0%	100.0%	83.1%	97.3%
UNC1999	95.4%	97.2%	99.9%	99.2%	100.0%	81.4%	97.5%	100.0%	99.0%	97.2%	90.9%	90.9%	100.0%	98.2%	92.0%
Valproic Acid (sodium salt)	100.0%	100.0%	93.3%	85.8%	100.0%	100.0%	100.0%	91.5%	100.0%	96.5%	84.9%	96.2%	88.3%	98.7%	100.0%
WDR5-0103	100.0%	97.7%	98.4%	98.8%	99.5%	100.0%	100.0%	100.0%	100.0%	99.1%	92.5%	100.0%	96.2%	100.0%	98.2%
Zebularine	100.0%	100.0%	93.3%	94.3%	100.0%	92.2%	98.5%	100.0%	99.4%	98.8%	100.0%	88.4%	79.8%	84.5%	100.0%

Compound	J19-2	J19-3	J19-4	J19-5	J19-6	J19-7	J2-2	J21-4	J21-5	J21-7	J4-1	J4-4	J7-6	J8-3	J8-4	J9-3	JEN
(-)-JQ1	100.0%	99.1%	100.0%	92.5%	93.8%	100.0%	100.0%	100.0%	100.0%	94.6%	95.8%	100.0%	100.0%	100.0%	92.1%	100.0%	97.6%
(-)-Neplanocin A	99.6%	99.5%	91.1%	83.4%	98.5%	100.0%	96.0%	100.0%	100.0%	100.0%	100.0%	86.3%	100.0%	100.0%	95.2%	98.2%	85.9%
(+)-Abscisic Acid	100.0%	92.0%	100.0%	100.0%	87.8%	92.6%	96.8%	100.0%	91.5%	89.0%	100.0%	100.0%	100.0%	100.0%	87.4%	95.9%	100.0%
(+)-JQ1	75.8%	100.0%	100.0%	79.2%	87.0%	79.0%	69.6%	86.1%	57.0%	97.9%	94.6%	59.9%	100.0%	57.6%	81.4%	74.5%	89.2%
(R)-PFI-2 (hydrochloride)	100.0%	88.1%	84.0%	100.0%	100.0%	100.0%	97.0%	100.0%	100.0%	97.9%	100.0%	100.0%	98.5%	100.0%	100.0%	100.0%	77.3%
1-Naphthoic Acid	100.0%	100.0%	100.0%	100.0%	84.4%	100.0%	98.9%	98.8%	100.0%	93.7%	100.0%	100.0%	100.0%	100.0%	90.6%	99.9%	90.9%
2-hexyl-4-Pentynoic Acid	100.0%	93.0%	98.9%	100.0%	100.0%	100.0%	97.2%	100.0%	100.0%	99.8%	100.0%	100.0%	98.8%	100.0%	100.0%	100.0%	98.3%
2-PCPA (hydrochloride)	100.0%	89.0%	100.0%	100.0%	100.0%	96.7%	99.7%	100.0%	97.4%	97.2%	97.3%	96.5%	87.7%	100.0%	100.0%	100.0%	97.1%
2,4-Pyridinedicarboxylic Acid	100.0%	100.0%	100.0%	82.3%	89.2%	100.0%	98.9%	100.0%	100.0%	100.0%	98.3%	100.0%	92.0%	98.2%	100.0%	98.3%	95.2%
2',3',5'-triacetyl-5-Azacytidine	100.0%	100.0%	100.0%	95.3%	88.5%	99.0%	95.3%	100.0%	98.7%	100.0%	100.0%	96.0%	100.0%	100.0%	99.0%	88.9%	100.0%
3-amino Benzamide	98.4%	95.9%	94.4%	97.9%	95.5%	90.4%	100.0%	99.9%	100.0%	91.3%	100.0%	100.0%	100.0%	100.0%	95.5%	97.7%	100.0%
3-Deazaneplanocin A	100.0%	79.4%	93.5%	100.0%	97.9%	84.3%	90.3%	90.9%	100.0%	97.5%	100.0%	78.5%	86.2%	100.0%	95.7%	98.1%	83.0%
3-Deazaneplanocin A (hydrochloride)	100.0%	94.1%	94.1%	100.0%	93.6%	92.2%	100.0%	97.2%	100.0%	94.3%	100.0%	81.4%	92.9%	99.3%	96.6%	96.0%	81.5%
3,3'-Diindolylmethane	100.0%	96.0%	93.7%	100.0%	100.0%	90.2%	89.2%	100.0%	100.0%	99.0%	100.0%	96.6%	95.6%	99.6%	98.6%	97.9%	100.0%
4-iodo-SAHA	96.7%	86.5%	81.7%	93.0%	100.0%	94.8%	92.5%	97.2%	89.2%	100.0%	100.0%	85.4%	67.5%	88.0%	81.1%	75.1%	90.8%
5-Azacytidine	100.0%	100.0%	100.0%	94.4%	94.8%	93.7%	95.5%	98.7%	100.0%	98.6%	96.1%	100.0%	100.0%	100.0%	88.5%	97.4%	99.3%
5-Methyl-2'-deoxycytidine	100.0%	89.8%	96.0%	100.0%	100.0%	100.0%	96.9%	100.0%	100.0%	95.7%	94.8%	100.0%	98.2%	100.0%	94.2%	100.0%	97.8%
5-Methylcytidine	100.0%	93.3%	100.0%	100.0%	99.3%	98.5%	98.8%	100.0%	100.0%	97.5%	100.0%	100.0%	100.0%	100.0%	100.0%	100.0%	100.0%
6-Thioguanine	80.5%	92.2%	82.2%	94.6%	87.6%	81.7%	95.4%	92.0%	72.8%	99.2%	100.0%	62.1%	83.6%	100.0%	83.2%	93.1%	48.3%
a-Hydroxyglutaric Acid (sodium salt)	100.0%	100.0%	83.3%	100.0%	99.3%	100.0%	87.8%	100.0%	100.0%	100.0%	100.0%	100.0%	97.2%	100.0%	100.0%	98.4%	96.8%
AGK2	100.0%	100.0%	83.3%	100.0%	100.0%	100.0%	100.0%	100.0%	100.0%	98.8%	100.0%	100.0%	100.0%	97.8%	95.9%	89.1%	96.0%
AGK7	100.0%	97.4%	100.0%	100.0%	99.3%	100.0%	100.0%	100.0%	100.0%	100.0%	91.9%	100.0%	100.0%	100.0%	100.0%	100.0%	95.6%
AK-7	100.0%	85.2%	90.4%	100.0%	95.3%	92.0%	99.5%	100.0%	100.0%	89.7%	89.6%	100.0%	99.9%	97.5%	100.0%	100.0%	99.4%
Anacardic Acid	100.0%	100.0%	86.8%	100.0%	100.0%	94.3%	93.8%	97.7%	97.0%	96.2%	96.8%	100.0%	100.0%	97.5%	100.0%	99.5%	98.7%
Apicidin	99.9%	58.8%	67.4%	67.0%	69.6%	92.4%	75.3%	93.9%	76.6%	96.3%	100.0%	52.7%	85.3%	86.4%	100.0%	71.9%	56.4%
BIX01294 (hydrochloride hydrate)	100.0%	100.0%	91.4%	100.0%	100.0%	100.0%	98.7%	95.6%	89.1%	98.5%	99.5%	24.0%	63.0%	100.0%	90.6%	76.5%	98.6%
BML-210	100.0%	89.8%	92.8%	97.8%	100.0%	100.0%	91.6%	96.2%	100.0%	90.1%	100.0%	100.0%	96.0%	96.9%	100.0%	99.7%	100.0%
BRD73954	98.4%	95.3%	94.4%	99.4%	97.1%	100.0%	91.3%	93.8%	97.9%	98.4%	94.1%	100.0%	92.6%	88.6%	79.2%	92.9%	100.0%
Bromosporine	93.7%	96.9%	100.0%	100.0%	98.0%	96.2%	74.7%	97.2%	73.7%	99.7%	93.7%	86.0%	82.0%	58.6%	61.3%	88.0%	97.7%
BSI-201	100.0%	100.0%	100.0%	91.9%	97.0%	92.2%	98.4%	95.0%	100.0%	95.1%	100.0%	100.0%	100.0%	100.0%	86.6%	100.0%	96.9%
Butyrolactone 3	98.9%	100.0%	92.7%	100.0%	91.0%	93.8%	92.4%	95.9%	92.1%	93.5%	95.6%	100.0%	100.0%	100.0%	100.0%	99.1%	99.6%
C646	100.0%	96.2%	93.0%	89.6%	97.1%	100.0%	98.5%	97.4%	100.0%	93.0%	100.0%	97.4%	100.0%	100.0%	82.5%	98.4%	100.0%
CAY10398	96.9%	72.2%	93.6%	87.3%	84.0%	100.0%	91.1%	88.6%	92.7%	94.9%	100.0%	56.2%	66.3%	80.6%	77.3%	87.5%	65.8%
CAY10591	100.0%	88.4%	100.0%	85.6%	100.0%	100.0%	96.8%	94.6%	100.0%	97.7%	100.0%	100.0%	100.0%	100.0%	100.0%	100.0%	84.3%
CAY10603	97.0%	77.1%	81.8%	100.0%	81.4%	100.0%	100.0%	94.1%	91.7%	99.0%	94.0%	80.3%	82.3%	89.9%	100.0%	95.6%	78.3%
CAY10669	100.0%	100.0%	92.1%	92.5%	92.2%	100.0%	97.8%	100.0%	100.0%	96.7%	96.9%	100.0%	100.0%	96.0%	100.0%	97.7%	95.7%
CAY10683	100.0%	98.6%	98.4%	100.0%	91.6%	100.0%	98.5%	96.1%	100.0%	100.0%	95.4%	100.0%	100.0%	100.0%	100.0%	99.5%	96.1%
CBHA	100.0%	93.7%	93.7%	100.0%	87.8%	89.5%	100.0%	100.0%	100.0%	100.0%	93.2%	94.5%	80.1%	100.0%	100.0%	94.8%	84.1%
CCG-100602	100.0%	100.0%	100.0%	89.6%	97.7%	99.6%	100.0%	100.0%	100.0%	97.9%	100.0%	100.0%	94.2%	100.0%	100.0%	99.6%	96.6%
Chidamide	100.0%	80.7%	94.3%	100.0%	95.5%	100.0%	90.4%	100.0%	88.3%	92.6%	92.9%	100.0%	98.6%	88.0%	100.0%	100.0%	100.0%
CI-994	95.4%	94.7%	93.1%	92.4%	100.0%	100.0%	89.7%	99.7%	95.3%	94.4%	100.0%	89.6%	88.1%	88.1%	75.8%	98.7%	89.0%
Cl-Amidine (hydrochloride)	100.0%	100.0%	91.6%	87.4%	88.3%	90.8%	100.0%	100.0%	100.0%	100.0%	97.7%	100.0%	100.0%	100.0%	97.9%	100.0%	100.0%
coumarin-SAHA	100.0%	84.9%	98.9%	100.0%	74.3%	80.8%	87.6%	93.2%	76.1%	100.0%	98.6%	76.1%	62.8%	99.1%	100.0%	91.7%	90.7%
CPI-203	80.3%	100.0%	100.0%	96.5%	100.0%	95.1%	61.1%	87.3%	51.9%	95.1%	85.9%	53.9%	68.0%	61.4%	68.2%	84.9%	86.4%
CPTH2 (hydrochloride)	100.0%	95.1%	93.7%	97.1%	85.8%	89.4%	98.0%	86.0%	77.1%	90.8%	98.3%	92.1%	83.5%	87.9%	74.3%	99.1%	97.1%
CUDC-101	100.0%	71.5%	86.7%	88.6%	96.2%	80.9%	65.7%	99.5%	87.0%	100.0%	100.0%	82.3%	57.9%	82.8%	100.0%	91.1%	95.4%
Daminozide	100.0%	94.1%	99.2%	82.4%	93.3%	98.8%	97.3%	100.0%	100.0%	95.5%	100.0%	100.0%	100.0%	100.0%	90.0%	100.0%	99.5%
Decitabine	100.0%	93.2%	93.3%	100.0%	95.1%	100.0%	100.0%	99.7%	98.2%	100.0%	100.0%	90.9%	92.6%	99.4%	94.6%	96.0%	100.0%
Delphinidin (chloride)	100.0%	100.0%	88.5%	87.2%	100.0%	100.0%	98.1%	100.0%	100.0%	94.6%	97.4%	100.0%	100.0%	94.5%	95.5%	98.7%	100.0%
DMOG	96.1%	99.1%	89.4%	98.3%	92.1%	100.0%	90.4%	99.4%	96.7%	100.0%	100.0%	95.0%	100.0%	97.4%	88.0%	98.4%	94.0%
Ellagic Acid	100.0%	96.8%	98.8%	100.0%	77.8%	94.3%	96.4%	96.0%	73.8%	97.5%	95.3%	96.2%	98.1%	100.0%	98.0%	98.6%	100.0%
EPZ005687	100.0%	100.0%	100.0%	100.0%	100.0%	87.4%	99.7%	100.0%	96.2%	100.0%	97.0%	98.9%	100.0%	100.0%	100.0%	100.0%	99.2%
EPZ5676	100.0%	99.8%	82.8%	100.0%	96.1%	100.0%	86.0%	100.0%	100.0%	99.8%	100.0%	100.0%	98.7%	100.0%	100.0%	100.0%	94.9%
Etoposide	96.0%	94.1%	93.9%	100.0%	77.1%	100.0%	92.5%	96.2%	81.0%	95.0%	100.0%	96.5%	100.0%	90.9%	85.9%	94.1%	97.2%
EX-527	100.0%	87.5%	88.1%	80.2%	100.0%	100.0%	86.3%	97.3%	100.0%	96.9%	100.0%	100.0%	99.9%	100.0%	85.7%	96.1%	100.0%
F-Amidine (trifluoroacetate salt)	100.0%	100.0%	89.8%	92.4%	94.4%	100.0%	98.3%	100.0%	100.0%	97.8%	99.4%	100.0%	100.0%	100.0%	91.2%	100.0%	100.0%
Garcinol	97.9%	97.3%	89.3%	96.3%	93.9%	100.0%	100.0%	100.0%	94.6%	94.5%	100.0%	99.4%	100.0%	100.0%	100.0%	84.2%	98.8%
Gemcitabine	44.7%	68.1%	72.6%	55.3%	72.2%	55.4%	76.2%	69.4%	71.4%	94.8%	100.0%	46.9%	88.1%	73.1%	55.9%	86.7%	61.0%
GSK-J1 (sodium salt)	100.0%	97.1%	100.0%	87.9%	93.6%	100.0%	97.8%	100.0%	100.0%	97.3%	100.0%	98.1%	100.0%	100.0%	87.0%	95.2%	97.5%
GSK-J2 (sodium salt)	98.7%	90.0%	100.0%	79.2%	91.2%	97.2%	92.4%	96.7%	100.0%	91.7%	100.0%	100.0%	98.8%	95.6%	78.3%	96.5%	97.4%
GSK-J4 (hydrochloride)	98.0%	100.0%	100.0%	94.2%	98.8%	96.6%	93.0%	100.0%	98.0%	93.2%	100.0%	99.3%	100.0%	100.0%	84.7%	92.6%	100.0%
GSK-LSD1 (hydrochloride)	100.0%	89.8%	97.1%	100.0%	100.0%	100.0%	90.3%	96.0%	100.0%	99.8%	100.0%	100.0%	100.0%	100.0%	100.0%	100.0%	100.0%
GSK126	100.0%	100.0%	94.7%	100.0%	90.4%	100.0%	95.8%	99.3%	100.0%	98.0%	98.9%	100.0%	97.3%	99.3%	100.0%	99.8%	76.6%
GSK2801	100.0%	93.4%	95.5%	100.0%	100.0%	100.0%	97.5%	96.5%	100.0%	97.7%	100.0%	96.1%	86.6%	100.0%	99.1%	100.0%	99.8%
GSK343	100.0%	87.5%	93.2%	100.0%	93.0%	100.0%	98.1%	100.0%	100.0%	99.1%	99.5%	95.0%	97.7%	100.0%	100.0%	98.8%	100.0%
GSK4112	100.0%	94.8%	100.0%	87.8%	90.0%	94.4%	98.8%	97.7%	100.0%	91.1%	100.0%	100.0%	100.0%	100.0%	78.3%	98.0%	100.0%
HC Toxin	47.1%	49.9%	62.1%	54.4%	32.1%	34.2%	63.8%	76.0%	57.2%	93.0%	100.0%	28.2%	28.7%	65.9%	90.7%	43.8%	27.7%
HNHA	100.0%	97.0%	100.0%	96.6%	91.0%	100.0%	98.5%	95.7%	90.7%	99.5%	100.0%	99.6%	86.5%	98.5%	100.0%	100.0%	100.0%
HPOB	100.0%	89.3%	100.0%	100.0%	97.9%	89.8%	97.8%	100.0%	100.0%	96.2%	98.4%	100.0%	99.2%	100.0%	100.0%	99.5%	97.8%
I-BET151	86.8%	94.1%	100.0%	100.0%	97.2%	90.1%	77.8%	97.1%	79.9%	94.5%	100.0%	62.9%	81.0%	77.3%	65.2%	86.7%	100.0%
I-BET762	84.6%	100.0%	100.0%	93.0%	100.0%	97.6%	76.3%	87.6%	68.7%	96.9%	100.0%	61.0%	94.6%	68.3%	85.2%	80.1%	100.0%
I-CBP112 (hydrochloride)	100.0%	100.0%	96.7%	100.0%	95.2%	92.8%	92.7%	100.0%	92.1%	100.0%	100.0%	100.0%	95.0%	99.3%	100.0%	100.0%	96.0%
IOX1	99.4%	99.4%	86.9%	84.5%	96.9%	100.0%	92.5%	100.0%	100.0%	97.2%	94.3%	100.0%	100.0%	100.0%	87.1%	97.4%	100.0%
Isoliquiritigenin	100.0%	100.0%	90.4%	100.0%	97.0%	90.3%	99.5%	100.0%	100.0%	97.8%	100.0%	100.0%	100.0%	100.0%	100.0%	100.0%	99.1%
ITF 2357	100.0%	75.8%	93.4%	98.3%	100.0%	99.7%	82.3%	100.0%	92.1%	100.0%	99.1%	72.2%	82.8%	100.0%	87.8%	73.5%	77.2%
JGB1741	97.5%	100.0%	92.9%	90.6%	94.1%	87.2%	100.0%	100.0%	100.0%	100.0%	100.0%	92.3%	100.0%	100.0%	100.0%	94.9%	100.0%
JIB-04	77.9%	35.7%	32.8%	73.4%	30.5%	35.8%	81.6%	77.5%	80.5%	99.8%	93.5%	93.9%	51.1%	81.1%	43.9%	100.0%	98.3%
LAQ824	43.7%	43.3%	44.3%	42.5%	38.0%	34.3%	38.5%	71.7%	55.7%	92.0%	96.6%	31.0%	77.9%	54.9%	21.4%	46.0%	54.6%
Lestaurtinib	63.9%	73.9%	82.9%	100.0%	62.4%	81.2%	63.0%	90.5%	49.5%	89.7%	99.8%	69.7%	97.5%	87.7%	98.7%	86.2%	100.0%
Lomeguatrib	100.0%	88.1%	100.0%	95.3%	94.1%	87.4%	94.5%	96.9%	98.4%	89.7%	100.0%	100.0%	100.0%	95.9%	79.3%	97.5%	100.0%
M 344	100.0%	88.2%	86.6%	100.0%	85.5%	88.7%	90.6%	97.8%	78.5%	100.0%	96.7%	67.1%	74.2%	100.0%	92.3%	83.0%	76.2%
MC 1568	100.0%	98.3%	99.5%	100.0%	100.0%	100.0%	100.0%	98.8%	100.0%	97.3%	100.0%	98.8%	95.6%	97.8%	96.0%	100.0%	93.6%
Methylstat (hydrate)	100.0%	98.9%	99.3%	100.0%	92.1%	96.7%	98.3%	100.0%	100.0%	93.5%	100.0%	100.0%	100.0%	99.7%	88.5%	96.5%	98.6%
MI-2 (hydrochloride)	97.8%	99.3%	98.4%	84.9%	100.0%	100.0%	99.0%	100.0%	99.2%	96.7%	97.4%	100.0%	100.0%	99.6%	87.8%	100.0%	100.0%
MI-nc (hydrochloride)	100.0%	86.6%	96.7%	97.2%	92.2%	92.0%	96.0%	91.3%	100.0%	90.4%	100.0%	73.6%	63.6%	89.4%	77.7%	96.9%	78.7%
Mirin	100.0%	95.8%	100.0%	100.0%	86.7%	100.0%	94.6%	93.6%	90.6%	100.0%	100.0%	96.7%	96.8%	100.0%	98.0%	94.3%	100.0%
MS-275	100.0%	90.7%	96.6%	100.0%	98.1%	91.5%	85.6%	94.2%	81.5%	100.0%	98.5%	86.6%	94.9%	100.0%	100.0%	93.4%	90.0%
N-Oxalylglycine	100.0%	100.0%	94.1%	100.0%	96.6%	100.0%	100.0%	97.8%	91.6%	95.2%	97.3%	99.5%	86.5%	100.0%	100.0%	100.0%	97.6%
Nicotinamide	100.0%	100.0%	96.6%	100.0%	97.6%	89.8%	98.8%	99.2%	100.0%	100.0%	100.0%	100.0%	100.0%	100.0%	100.0%	100.0%	97.6%
Octyl-a-ketoglutarate	100.0%	95.4%	100.0%	90.6%	86.3%	100.0%	100.0%	97.7%	100.0%	96.3%	100.0%	100.0%	95.3%	100.0%	79.5%	100.0%	99.9%
OTX015	88.2%	100.0%	100.0%	100.0%	90.4%	92.0%	61.1%	89.4%	70.5%	90.9%	88.8%	64.2%	95.0%	51.1%	100.0%	85.5%	98.3%
Oxamflatin	100.0%	87.0%	98.4%	100.0%	81.4%	94.9%	92.3%	97.2%	90.3%	100.0%	92.9%	74.2%	95.8%	100.0%	75.9%	96.0%	77.5%
Panobinostat	28.5%	52.4%	48.4%	37.4%	32.8%	25.5%	42.3%	50.0%	36.0%	52.7%	99.1%	29.6%	82.9%	57.0%	69.9%	42.2%	64.1%
PCI 34051	96.2%	91.8%	87.2%	89.2%	94.8%	100.0%	97.8%	98.9%	99.9%	96.6%	100.0%	98.0%	96.5%	100.0%	100.0%	100.0%	100.0%
PFI-1	100.0%	100.0%	100.0%	89.8%	100.0%	100.0%	90.0%	96.2%	87.6%	100.0%	89.3%	89.4%	65.0%	96.0%	64.7%	92.0%	96.0%
PFI-3	100.0%	88.9%	100.0%	100.0%	95.6%	97.7%	100.0%	100.0%	100.0%	100.0%	96.7%	100.0%	94.9%	100.0%	100.0%	95.7%	96.9%
Phthalazinone pyrazole	100.0%	100.0%	100.0%	96.3%	94.4%	97.6%	100.0%	100.0%	100.0%	91.6%	100.0%	100.0%	73.3%	98.3%	100.0%	92.0%	70.1%
Piceatannol	100.0%	96.3%	99.7%	92.1%	100.0%	100.0%	93.9%	100.0%	100.0%	95.0%	100.0%	100.0%	92.1%	89.5%	100.0%	100.0%	100.0%
Pimelic Diphenylamide 106	100.0%	100.0%	94.2%	100.0%	100.0%	96.1%	93.4%	95.9%	94.5%	94.8%	100.0%	100.0%	100.0%	100.0%	100.0%	97.0%	74.1%
Pyroxamide	100.0%	92.6%	93.5%	100.0%	91.0%	97.8%	91.9%	98.6%	90.9%	100.0%	100.0%	76.4%	86.2%	100.0%	100.0%	94.6%	89.0%
RG-108	100.0%	100.0%	100.0%	100.0%	90.6%	88.1%	99.2%	100.0%	88.4%	100.0%	99.9%	100.0%	99.7%	100.0%	100.0%	90.4%	100.0%
RGFP966	100.0%	89.7%	100.0%	100.0%	100.0%	100.0%	89.7%	99.0%	100.0%	100.0%	96.6%	100.0%	95.9%	100.0%	95.8%	100.0%	95.6%
RSC-133	100.0%	83.2%	92.7%	96.8%	98.8%	88.1%	92.6%	98.8%	100.0%	93.7%	100.0%	100.0%	99.8%	93.2%	100.0%	100.0%	100.0%
Rucaparib (phosphate)	100.0%	93.0%	100.0%	81.6%	94.9%	99.9%	96.4%	100.0%	100.0%	94.9%	100.0%	100.0%	95.5%	99.9%	74.7%	100.0%	87.3%
RVX-208	100.0%	88.6%	97.5%	100.0%	89.8%	100.0%	95.7%	89.5%	96.2%	95.3%	100.0%	94.1%	93.0%	84.8%	90.1%	100.0%	100.0%
S-(5'-Adenosyl)-L-methionine (tosylate)	99.8%	100.0%	100.0%	100.0%	100.0%	100.0%	92.3%	100.0%	100.0%	97.3%	100.0%	100.0%	100.0%	100.0%	98.6%	100.0%	78.2%
S-Adenosylhomocysteine	100.0%	84.0%	99.3%	100.0%	88.6%	100.0%	100.0%	94.6%	90.8%	99.7%	100.0%	100.0%	99.2%	88.9%	97.4%	98.0%	89.9%
SAHA	100.0%	77.5%	92.3%	100.0%	93.9%	100.0%	92.8%	96.3%	90.8%	95.4%	99.3%	48.6%	65.0%	100.0%	90.8%	90.8%	65.5%
Salermide	100.0%	95.7%	100.0%	100.0%	85.0%	94.2%	100.0%	93.2%	87.2%	96.9%	93.7%	100.0%	93.5%	100.0%	71.5%	100.0%	97.5%
SB939	100.0%	69.3%	77.5%	91.0%	83.4%	100.0%	75.2%	97.1%	73.8%	93.0%	100.0%	34.5%	72.9%	98.7%	93.3%	63.3%	41.4%
Scriptaid	100.0%	83.4%	92.7%	100.0%	88.9%	94.2%	100.0%	90.4%	94.1%	99.3%	100.0%	100.0%	86.1%	100.0%	86.7%	85.6%	76.6%
SGC-CBP30	98.7%	100.0%	100.0%	93.4%	100.0%	96.8%	96.6%	100.0%	99.2%	98.7%	95.9%	100.0%	100.0%	100.0%	100.0%	94.8%	97.8%
SGC0946	100.0%	86.0%	87.3%	100.0%	88.1%	100.0%	99.4%	90.3%	97.7%	99.7%	100.0%	99.9%	92.5%	90.8%	100.0%	100.0%	100.0%
SGI-1027	95.7%	90.9%	100.0%	83.2%	88.6%	96.4%	99.3%	100.0%	100.0%	98.5%	97.9%	100.0%	90.3%	99.3%	100.0%	100.0%	94.8%
Sinefungin	100.0%	82.6%	87.0%	100.0%	100.0%	100.0%	100.0%	100.0%	100.0%	95.7%	100.0%	100.0%	88.9%	95.4%	100.0%	97.9%	85.3%
SIRT1/2 Inhibitor IV	100.0%	100.0%	98.2%	100.0%	100.0%	100.0%	96.2%	100.0%	100.0%	100.0%	97.8%	100.0%	100.0%	100.0%	100.0%	97.7%	95.8%
Sirtinol	100.0%	100.0%	86.8%	100.0%	91.0%	100.0%	97.3%	100.0%	99.9%	96.8%	100.0%	100.0%	94.5%	100.0%	100.0%	99.3%	100.0%
Sodium 4-Phenylbutyrate	100.0%	100.0%	100.0%	87.6%	89.4%	100.0%	93.9%	100.0%	100.0%	97.7%	96.8%	100.0%	100.0%	100.0%	94.5%	100.0%	95.6%
Sodium Butyrate	100.0%	99.3%	100.0%	100.0%	90.4%	87.5%	100.0%	94.5%	80.2%	94.6%	100.0%	96.7%	94.0%	90.6%	96.8%	100.0%	80.9%
Splitomicin	100.0%	95.9%	91.1%	100.0%	85.3%	100.0%	98.1%	95.6%	85.1%	99.5%	100.0%	98.9%	98.8%	100.0%	100.0%	100.0%	95.2%
Suramin (sodium salt)	100.0%	100.0%	98.3%	100.0%	96.5%	87.0%	92.1%	100.0%	100.0%	97.9%	100.0%	100.0%	100.0%	96.7%	100.0%	98.8%	97.7%
Tenovin-1	100.0%	96.4%	97.8%	100.0%	95.1%	79.8%	99.6%	95.0%	88.7%	96.1%	99.8%	100.0%	86.8%	94.0%	100.0%	95.2%	99.0%
Tenovin-6	100.0%	95.8%	100.0%	100.0%	93.6%	84.1%	93.1%	93.4%	87.2%	92.8%	100.0%	100.0%	100.0%	90.1%	87.5%	99.2%	96.8%
trans-Resveratrol	95.3%	94.0%	98.8%	99.5%	97.8%	100.0%	92.8%	91.5%	100.0%	98.3%	100.0%	95.2%	100.0%	98.7%	92.8%	100.0%	97.5%
Trichostatin A	95.9%	58.4%	73.5%	79.0%	77.7%	96.8%	67.6%	84.6%	80.1%	93.2%	99.9%	56.1%	75.2%	88.9%	68.9%	73.7%	69.8%
Tubacin	100.0%	89.8%	88.7%	100.0%	96.6%	98.3%	94.2%	100.0%	91.6%	100.0%	99.3%	99.4%	96.7%	95.8%	100.0%	100.0%	100.0%
Tubastatin A	100.0%	88.4%	93.5%	100.0%	87.9%	100.0%	100.0%	91.0%	100.0%	100.0%	100.0%	99.4%	93.1%	87.2%	100.0%	100.0%	100.0%
Tubastatin A (trifluoroacetate salt)	100.0%	100.0%	90.7%	94.2%	98.2%	100.0%	95.3%	100.0%	95.2%	98.8%	100.0%	99.4%	100.0%	100.0%	85.5%	95.7%	100.0%
UNC0224	100.0%	79.9%	88.9%	100.0%	99.3%	95.9%	95.2%	100.0%	88.2%	100.0%	100.0%	97.4%	87.8%	100.0%	37.9%	100.0%	100.0%
UNC0321 (trifluoroacetate salt)	96.8%	100.0%	92.9%	88.6%	91.4%	100.0%	100.0%	100.0%	100.0%	97.3%	100.0%	100.0%	100.0%	100.0%	84.9%	100.0%	98.0%
UNC0631	100.0%	81.7%	87.4%	100.0%	86.9%	100.0%	62.3%	88.3%	85.5%	96.1%	100.0%	33.2%	65.6%	54.9%	100.0%	70.2%	80.8%
UNC0638	100.0%	100.0%	97.4%	100.0%	93.0%	92.7%	100.0%	100.0%	100.0%	91.7%	100.0%	24.5%	36.3%	100.0%	70.0%	81.8%	97.7%
UNC0642	100.0%	100.0%	98.7%	100.0%	100.0%	88.4%	93.9%	100.0%	100.0%	100.0%	99.2%	58.6%	100.0%	100.0%	76.4%	100.0%	96.1%
UNC0646	97.1%	78.3%	92.0%	100.0%	87.1%	92.0%	95.9%	100.0%	100.0%	100.0%	100.0%	45.1%	36.4%	100.0%	84.4%	81.1%	56.6%
UNC1215	100.0%	84.0%	90.4%	100.0%	100.0%	94.0%	100.0%	100.0%	94.5%	97.5%	98.5%	100.0%	97.5%	100.0%	100.0%	100.0%	100.0%
UNC1999	100.0%	91.9%	89.9%	100.0%	93.0%	100.0%	95.7%	90.0%	100.0%	96.8%	100.0%	95.6%	97.1%	83.6%	100.0%	100.0%	100.0%
Valproic Acid (sodium salt)	100.0%	98.5%	91.1%	100.0%	90.5%	100.0%	100.0%	95.0%	92.0%	100.0%	100.0%	99.2%	100.0%	99.3%	92.7%	100.0%	100.0%
WDR5-0103	100.0%	100.0%	91.6%	100.0%	99.6%	93.5%	100.0%	100.0%	94.7%	100.0%	98.1%	100.0%	97.7%	100.0%	100.0%	96.1%	99.5%
Zebularine	100.0%	100.0%	98.1%	78.8%	90.8%	99.0%	98.6%	100.0%	100.0%	98.0%	100.0%	100.0%	100.0%	94.9%	92.4%	100.0%	96.4%

Supplementary Table S2. p value for each epigenetic compound and meningioma sample. Complete table of p values for each compound-sample pair. p values are calculated using the Mann-Whitney-U test (confidence level of 0.95).

Compound	CH157	GAR	H23-3	IOMM Lee	J10-6	J11-2	J11-6	J11-7	J13-2	J15-4	J16-5	J17-2	J17-7	J18-6	J18-7
(-)-JQ1	0.4600169	0.7624968	0.6411289	0.1616841	0.0741806	0.8776660	0.5380913	0.3520149	0.3616612	0.0935565	0.4234913	0.6664780	0.0453981	0.0068238	0.2717521
(-)-Neplanocin A	0.0563117	0.4402933	0.1112544	0.0008153	0.0177687	0.7348866	0.3719816	0.7582031	0.0416067	0.2674955	0.6007483	0.4234767	0.0193060	0.5168881	0.4137957
(+)-Abscisic Acid	0.9754417	0.7257595	0.6135839	0.2861859	0.1755719	0.1314384	0.6223225	0.0193047	0.4157133	0.0047426	0.3245839	0.8293827	0.0061484	0.0590141	0.6318908
(+)-JQ1	0.0336614	0.5636528	0.0097921	0.4336617	0.3719916	0.0964371	0.0038061	0.0038072	0.5811601	0.2294762	0.2952613	0.6223261	0.0046242	0.0037010	0.0037010
(R)-PFI-2 (hydrochloride)	0.1855969	0.3233694	0.0359454	0.8647421	0.0126476	0.8534576	0.1164059	0.1567550	0.2968109	0.0191125	0.1659695	0.3557242	0.4415401	0.2047554	0.0208442
1-Naphthoic Acid	0.0847239	0.2795449	0.3078981	0.6120164	0.0193035	0.4600075	0.5380913	0.8293862	0.5016720	0.0102497	0.2812868	1.0000000	0.0421809	0.0806050	0.8990885
2-hexyl-4-Pentynoic Acid	0.4415353	0.4569893	0.0433982	0.3331004	0.2546929	0.7817318	0.0163397	0.2420827	0.0141841	0.4094803	0.2181911	0.7348866	0.2314679	0.0485378	0.2256050
2-PCPA (hydrochloride)	0.9019990	0.2851412	0.7856959	0.4887152	0.4415305	0.5279788	0.6007331	0.2677631	0.5227660	0.3559394	0.4982513	0.4600075	0.0421809	0.2256050	0.0806050
2,4-Pyridinedicarboxylic Acid	0.0126485	0.2733032	0.4487824	0.5917198	0.3399227	0.5179684	1.0000000	0.6349004	0.4157133	0.0017164	0.0904347	0.1567464	0.0067491	0.0057505	0.1082115
2',3',5'-triacetyl-5-Azacytidine	0.9877193	0.1496235	0.8473414	0.1850215	0.0767064	0.4058670	0.3719816	0.3557343	0.0107449	0.5589062	0.6890170	0.9019970	0.1027761	0.1021613	0.1352900
3-amino Benzamide	0.5179772	0.7016080	0.5342742	0.0304738	0.9019980	0.6890077	0.9141948	0.0081105	0.0125509	0.0011102	0.2014112	0.8049681	0.0097142	0.1082115	0.9662827
3-Deazaneplanocin A	0.0288395	0.0813221	0.1136623	0.0004461	0.1960294	0.0421744	0.3719816	0.8054709	0.5227660	0.1261534	0.3557393	0.4507083	0.0067491	0.0087729	0.0908702
3-Deazaneplanocin A (hydrochloride)	0.4789236	0.6233695	0.1999014	0.0008909	0.1855923	0.0391526	0.5179641	0.6442521	0.2490982	0.0648541	0.5795046	0.0081092	0.0050893	0.0044241	0.6931928
3,3'-Diindolylmethane	0.1354252	0.3908694	0.0097921	0.2834093	0.0137874	0.0311670	0.9754409	0.5586160	0.0058075	0.0566054	0.5179815	0.3972237	0.0115938	0.0052731	0.0396698
4-iodo-SAHA	0.1479378	0.2901864	0.4844386	0.0008153	0.3557293	0.1899965	0.7348840	0.0088797	0.0000623	0.0097755	0.0741866	0.8293827	0.0055963	0.2365802	1.0000000
5-Azacytidine	0.0115929	0.3908694	0.2285332	0.0060811	0.7118196	0.9019970	0.0524453	0.5586160	0.9825683	0.0369239	0.9754420	0.2624373	0.0647430	0.1673279	0.4899264
5-Methyl-2'-deoxycytidine	0.1094268	0.1755709	0.5152861	0.2198928	0.3887070	0.6890077	0.0046225	0.6622175	0.0449995	0.0018832	0.5795046	0.6890077	0.1237578	0.1948723	0.6725096
5-Methylcytidine	0.3719966	0.5443144	0.1156822	0.0061078	0.0081098	0.6890077	0.0126458	0.1354252	0.0011086	0.0427119	0.0647430	0.7581982	0.0384258	0.1506607	0.4137957
6-Thioguanine	0.0038072	0.0794546	0.0223110	0.0008153	0.0038069	0.8293827	0.4507035	0.0563117	0.2917816	0.5962202	0.0081112	0.1237461	0.0067491	0.0037010	0.0037010
a-Hydroxyglutaric Acid (sodium salt)	0.7118225	0.8417386	0.2123457	0.3914892	0.4324467	0.0106146	0.6007331	0.3096883	0.6502825	0.9651447	0.1395088	0.0488126	0.9264188	0.2970985	0.5168881
AGK2	0.4789236	0.9451839	0.2123457	0.3361883	0.1855785	0.0391526	0.0067468	0.9386545	0.1571470	0.6700637	0.0904347	0.5794927	0.0185234	0.0396698	0.2537383
AGK7	0.1094268	0.4151468	0.3927975	0.0895241	0.0074009	0.1394964	0.1659519	0.4415353	0.0492495	0.0318182	0.7348946	0.3557242	0.1479420	0.0300150	0.5539920
AK-7	0.6223334	0.4089953	0.7952708	0.6248112	0.0193035	0.2952462	0.0193009	0.1436015	0.0640667	0.0037259	0.0074021	0.8293827	0.3096934	0.0424593	0.3523949
Anacardic Acid	0.2546978	0.7641575	0.0204216	0.9165320	0.0847206	0.2812717	0.1094157	0.5381041	0.7555343	0.4878621	0.5795046	0.6664780	0.0246261	0.0121397	0.1021613
Apicidin	0.0038072	0.2372727	0.0103573	0.0008153	0.3887070	0.2013971	0.4058621	0.1659651	0.1094988	0.0002929	0.6442555	0.2546879	0.0061484	0.0037010	0.0806050
BIX01294 (hydrochloride hydrate)	0.0038072	0.1166750	0.0029647	0.0229153	0.1855923	0.0847174	0.3096732	0.0050888	0.0262056	0.1107131	0.4415401	0.2812717	0.1237578	0.3823399	0.7673089
BML-210	0.1755764	0.3987689	0.0804721	0.5492127	0.3399227	0.5586078	0.0647319	1.0000000	0.1772776	0.0786004	0.1960388	0.7581982	0.2068998	0.0142184	0.2970985
BRD73954	0.6007445	0.8375200	0.1384725	0.1103311	0.6442486	0.8898193	0.8534561	0.6442521	0.3674409	0.1144262	0.3399328	0.0524477	0.2677681	0.1352900	0.5730206
Bromosporine	0.9509067	0.3449726	0.0831658	0.5581009	0.3557293	0.1149092	0.2298992	0.4361334	0.2968109	0.0389462	0.2068998	0.0421744	0.2812868	0.0044241	0.0037010
BSI-201	0.1855969	0.3647626	1.0000000	0.0068020	0.0323918	0.4982379	0.8054649	0.4600169	0.2190664	0.0056161	0.9754420	0.1479294	0.0074021	0.0044241	0.2149981
Butyrolactone 3	0.3887120	0.9960128	0.0117557	0.0252305	0.0097127	0.7118167	0.2546830	0.2677631	0.6820469	0.4033051	0.2299186	0.9631687	0.0336632	0.1280735	0.0781892
C646	0.1027725	0.4999546	0.0209570	0.1580207	0.5794967	0.0819719	1.0000000	0.5179772	0.5515833	0.0010682	0.2420876	0.1488617	0.6890170	0.0134941	0.4289809
CAY10398	0.0038072	0.0832249	0.0112084	0.0008524	0.4058719	0.1960248	0.0363147	0.0041975	0.1772776	0.1248063	0.8534621	0.2068856	0.0563142	0.0087729	0.0037010
CAY10591	0.1960341	0.3561116	0.4966566	0.7292101	0.0193035	0.9509056	0.4234719	0.5381041	0.0640667	0.2628105	0.7817384	0.5794927	1.0000000	0.0112051	0.6725095
CAY10603	0.0038072	0.0706339	0.3704674	0.0007798	0.6223297	0.0163409	0.3245536	0.0150179	0.0003926	0.0005449	0.8293879	0.0163409	0.0067491	0.3239787	0.7352388
CAY10669	0.4058768	0.2372727	0.5601281	0.7814576	0.8414016	0.8054669	0.8534561	0.3399278	0.5886686	0.2446382	0.7118254	0.9509056	0.0847271	0.0259972	0.4137957
CAY10683	0.2299138	0.8248930	0.4872331	0.4937840	0.1755719	0.3719766	0.4415210	0.6223334	0.0689204	0.0421556	0.1027761	0.2466041	0.5179815	0.9775183	0.0424593
CBHA	0.2952563	0.5780075	0.0301701	0.0170414	0.6223297	0.5794927	0.3557192	0.0391567	0.0050763	0.8441067	0.0647430	0.0453846	0.0115938	0.0193367	0.0044241
CCG-100602	0.1237539	0.3264282	0.3981965	0.5174291	0.0115921	0.0453913	0.1164059	0.3557343	0.4094822	0.2917785	0.0177711	0.3887021	0.0209555	0.0590141	1.0000000
Chidamide	0.0246246	0.2276814	0.3125294	0.0425349	0.3557293	0.0106146	0.4058621	0.0336614	0.3674409	0.0416059	0.4982513	0.7581982	0.7118254	0.0713023	0.2047554
CI-994	0.0311705	0.1957136	0.4905260	0.0018503	0.8776710	0.6007369	0.5794888	0.0391567	0.0062077	0.4251619	0.8293879	0.5179684	0.5179815	0.0806050	0.3978805
Cl-Amidine (hydrochloride)	0.3245788	0.4089953	0.8841873	0.1076084	0.4415305	0.1855877	0.1567421	0.7582031	0.5370775	0.1131775	0.7348946	0.8942263	0.1237578	0.4300817	0.0279439
coumarin-SAHA	0.7348919	0.6233695	0.0123266	0.0121036	0.3024049	0.0227222	0.0097112	0.0669999	0.0073187	0.2316008	0.0488196	0.3802829	0.0055963	0.1021613	0.0037010
CPI-203	0.8898215	0.4597133	0.0029647	0.0478239	0.0038069	0.9019970	0.0336558	0.0038072	0.1917272	0.0531550	0.0038076	0.1855877	0.0061484	0.0037010	0.0037010
CPTH2 (hydrochloride)	0.8293862	0.9253014	0.8212076	0.3648094	0.2181815	0.0256203	0.8534561	0.1164173	0.0524877	0.1144253	0.4982513	0.0311670	0.0067491	0.0087729	0.0279439
CUDC-101	0.0081105	0.1436183	0.0092387	0.6526838	0.3557293	0.1567464	0.2812667	0.0038072	0.3851187	0.0601127	0.9509072	0.3926993	0.3720016	0.0628986	0.0074254
Daminozide	0.4600169	0.2372727	0.5342742	0.0671047	0.0336595	0.0600521	0.3972040	0.5381041	0.7721979	0.0096217	0.0074021	0.2677531	0.5308014	0.7565726	0.8657317
Decitabine	0.8293862	0.8039497	0.0546356	0.4860328	0.3719916	0.3719866	0.0150148	0.0084866	0.0146206	0.2628105	0.0177711	0.0227222	0.0081112	0.1746902	0.0050715
Delphinidin (chloride)	0.0453958	0.2780570	0.9635374	0.0748058	0.3887070	0.5380956	1.0000000	1.0000000	0.7143844	0.9782113	0.2547028	0.8293827	0.0126495	0.1352900	0.0279439
DMOG	0.4982468	0.9655645	0.7440968	0.0536551	0.0377119	0.1612973	0.7348840	0.7348919	0.0461811	0.1447203	0.2677681	0.4415258	0.1960388	0.0121397	0.6725095
Ellagic Acid	0.1855969	0.7257592	0.0924942	0.7068736	0.8776710	0.1479294	0.0055943	0.0209642	0.6780436	0.3336298	0.8054729	0.0106146	0.0061484	0.4637469	0.0300150
EPZ005687	0.2181863	0.4276124	0.5246934	0.2293040	0.4982424	0.1522850	0.8534561	0.0773274	0.6038185	0.3791673	0.8293879	0.1064202	0.7118254	0.7673089	0.2365802
EPZ5676	0.9754417	0.7831465	0.5579381	0.6262397	0.3719916	0.2744505	0.6223225	0.5381041	0.9738550	0.0005449	0.4415401	0.2420729	0.4058817	0.2970985	0.6931928
Etoposide	0.4982468	0.9269044	0.0719277	0.2861859	0.9509061	0.3557242	0.7817296	0.0793114	0.1653129	0.2402345	0.9754420	0.3557242	0.8054729	0.9775183	0.9775183
EX-527	0.1027725	0.4956765	0.1866094	0.1453541	0.3245738	0.1094194	0.0793020	0.1855969	0.3044623	0.0162426	0.9020000	0.6890077	0.9754420	0.0179254	0.8216344
F-Amidine (trifluoroacetate salt)	0.0311705	0.3449726	0.6976948	0.1769804	0.6223297	0.0318468	0.8776685	0.5586160	0.4283423	0.4542574	0.1567594	0.4704286	0.1755809	0.9214302	0.0166051
Garcinol	0.7118225	0.7977409	0.6135839	0.0125326	0.3887070	0.1286264	0.6664747	0.6664845	0.0020274	0.1477537	0.2812868	0.2181767	0.0421809	0.0856133	0.1352900
Gemcitabine	0.0038072	0.7420152	0.0359454	0.0947839	0.0421766	0.0038065	0.0038061	0.0038072	0.0000859	0.0007510	0.0038076	0.1164097	0.0046242	0.0037010	0.3671772
GSK-J1 (sodium salt)	0.1755764	0.2901864	0.8460059	0.8099964	0.1237500	0.7118167	0.0563039	0.1354252	0.8483846	0.0075610	0.0504271	0.3557242	0.1856015	0.0713023	0.0322167
GSK-J2 (sodium salt)	0.2624477	0.7420152	0.2724974	0.0947839	0.1567507	0.8054669	0.6777005	0.0288395	0.0384330	0.2170229	0.0847271	0.7348866	0.1856015	0.0590141	0.2479277
GSK-J4 (hydrochloride)	0.0647402	0.3129058	0.1347914	0.8872439	0.6223297	0.5690005	0.0391505	0.1542952	0.1094988	0.9782112	0.3557393	0.5380956	0.6442555	0.2596508	0.4137957
GSK-LSD1 (hydrochloride)	0.8776723	0.7217143	0.9845047	0.3041663	0.0074009	0.5794927	0.8776685	0.1027725	0.0283590	0.0070830	0.6223370	0.9243384	0.4789282	0.1082115	0.8436218
GSK126	0.1237539	0.6856710	0.4563100	0.0635060	0.6664813	0.3557242	0.5689964	0.9243401	0.7143851	0.0031256	0.4234913	0.4982379	0.3557393	0.0074254	0.4383586
GSK2801	0.4600169	0.2780570	0.9332942	0.7962073	0.7118196	0.0453913	0.0177663	0.1314465	0.3559413	0.0196794	0.8293879	0.7118167	0.0311723	0.2149981	0.0856133
GSK343	0.0177699	0.5952141	0.0178146	0.1782245	0.3096833	0.3399177	0.0793020	0.6622175	0.2231898	0.0247676	0.0421809	0.9509056	0.9264188	0.0087729	0.2098279
GSK4112	0.2068951	0.2205740	0.5732786	0.5917198	0.6442486	0.8776698	0.2677481	0.3399278	0.0427127	0.3175017	0.0604069	0.9754412	0.0209555	0.0074254	0.4137957
HC Toxin	0.0038072	0.1166750	0.0097921	0.0036438	0.0088790	0.8655465	0.0038061	0.0038072	0.0001345	0.0002808	0.0038076	0.0391526	0.0038076	0.0037010	0.0037010
HNHA	0.8534606	0.3617652	0.4462529	0.9165320	0.0336595	0.5483001	0.3557192	0.9019990	0.9346990	0.0091732	0.3557393	1.0000000	0.0163442	0.4988310	0.0142184
HPOB	0.7817362	0.3477359	0.3315825	0.2964736	0.0453936	0.0246216	0.0421723	0.4234864	0.0075613	0.1059117	0.3245839	0.1094194	0.1237578	0.0806050	0.2149981
I-BET151	0.0067486	0.9440708	0.1249522	0.5055333	0.3719916	0.1755674	1.0000000	0.4058768	0.0041347	0.8227926	0.8776736	0.0904246	0.0137894	0.0131428	0.0037010
I-BET762	0.0126485	0.6233695	0.0003242	0.5335120	0.6223297	0.0150158	0.0288345	0.0038072	0.0091737	0.1571454	0.0055963	0.7348866	0.0055963	0.0001575	0.0019655
I-CBP112 (hydrochloride)	1.0000000	0.6081846	0.2379284	0.0041481	0.6007407	0.4234767	0.3886971	0.6223334	0.6502825	0.4033051	0.6664878	0.3245687	0.6223370	0.7352388	0.9775183
IOX1	0.0847239	0.2976331	0.8918593	0.0344677	0.9019980	0.6890077	0.0311652	0.0266602	0.7721979	0.3616606	0.3887169	1.0000000	0.0067491	0.0424593	0.7780936
Isoliquiritigenin	0.8942286	0.2639630	0.3033150	0.5664180	0.0150169	0.6442452	0.1200263	0.6223334	0.1917272	0.8185446	0.6007483	0.9545594	0.0193060	0.0068238	0.0057505
ITF 2357	0.2611690	0.9651007	0.0122313	0.0334295	0.7118196	0.1960248	0.0693212	0.0847239	0.0024329	0.9912836	0.4982513	0.9019970	0.0115938	0.3239787	0.0131428
JGB1741	0.6223334	0.5443144	0.8157144	0.7625914	1.0000000	0.2068856	0.2677481	0.3096883	0.5962216	0.3559394	0.4694176	0.3557242	0.0563142	0.3309349	0.4811149
JIB-04	0.0193047	0.1294148	0.0846645	0.0325571	0.0377119	0.0038065	0.3557192	0.7348919	0.0580377	0.0000494	0.5795046	0.1659563	0.0391587	0.0037010	0.8216344
LAQ824	0.0038072	0.1166750	0.0031304	0.0005477	0.0038069	0.4234767	0.1479251	0.0038072	0.0000372	0.0003519	0.0038076	0.0061468	0.0038076	0.0037010	0.0037010
Lestaurtinib	0.4058768	0.7689298	0.0029647	0.1809678	0.7817340	0.0421744	0.2677481	0.2068951	0.0001604	0.2746296	0.0137894	0.3170611	0.0074021	0.0037010	0.0037010
Lomeguatrib	0.3245788	0.6387161	0.3219263	0.3914892	0.5179728	0.9141957	0.1755629	0.4234864	0.1508370	0.0017814	0.2812868	0.0563065	0.0074021	0.1948723	0.1021613
M 344	0.0038072	0.1166750	0.0135426	0.0689660	0.7348893	1.0000000	0.6442417	0.0391567	0.6268654	0.0005790	0.0266618	0.2677531	0.0088804	0.3103465	0.0040479
MC 1568	0.2420827	0.6310229	0.3442411	0.7625914	0.8293844	0.4982379	0.1027617	0.2952563	0.6581671	0.0318182	0.4789282	0.1094194	0.5381083	0.1021613	0.3671772
Methylstat (hydrate)	0.6223334	0.9784737	0.2854331	0.1870729	0.0741806	0.0067474	0.6442417	0.0177699	0.7555343	0.0002691	0.7582056	1.0000000	0.0453981	0.0131428	0.0057505
MI-2 (hydrochloride)	0.1164173	0.2165344	0.4146650	0.0324181	0.7817340	0.2677531	0.8054649	0.1128757	0.6038185	0.9607912	0.0194812	0.0693241	0.8776736	0.8657317	0.4467339
MI-nc (hydrochloride)	0.0038072	0.0706339	0.7952708	0.0007798	0.2181815	0.2546879	0.3477538	0.0126485	0.2402382	0.3703491	0.0246261	0.0266570	0.0055963	0.9214302	0.0057505
Mirin	0.6890139	0.2526868	0.7823849	0.4631792	0.8054689	0.8534576	0.3096732	0.2181863	0.0301186	0.0511709	0.5586201	0.0246216	0.1659695	0.2970985	0.2422035
MS-275	0.0038072	0.0954120	0.7823849	0.0008524	0.1060510	0.4982379	0.6442417	0.0074015	0.0374219	0.0001469	0.7118254	0.9754412	0.8054729	0.8990885	0.1021613
N-Oxalylglycine	0.9264181	0.3561116	0.1297765	0.9557529	0.6007407	0.3887021	0.9264158	0.7348919	0.7889666	0.9651447	1.0000000	0.0647346	0.3170763	0.0396698	0.0485378
Nicotinamide	0.0563117	0.6081846	0.4966566	0.7814576	0.0067480	0.4415258	0.7348840	0.9264181	0.6981475	0.0740710	0.7582056	0.5380956	0.0964475	0.0103349	0.2970985
Octyl-a-ketoglutarate	0.0177699	0.2165344	0.9767588	0.2806520	0.1659607	0.5586078	0.4415210	0.2546978	0.1808116	0.1720712	0.1755809	0.0524477	0.0074021	0.0518413	0.3379950
OTX015	0.0583255	0.3077586	0.1026448	0.7792948	0.1237500	0.0150158	0.8293809	0.0046238	0.2968109	0.9260154	0.0524551	0.7465109	0.1659695	0.0037010	0.0037010
Oxamflatin	0.0038072	0.1166750	0.0072141	0.0148864	0.6442486	0.4600075	0.1855831	0.0563117	0.4411917	0.0002173	0.6553301	0.1907491	0.0391587	0.0259972	0.0037010
Panobinostat	0.0038072	0.1043025	0.0029647	0.0029048	0.0038069	0.1394964	0.0038061	0.0038072	0.0000372	0.0001125	0.0038076	0.0038065	0.0038076	0.0037010	0.0037010
PCI 34051	0.4600169	0.7338725	0.5865737	0.5498437	0.9386538	0.7348866	0.1237422	0.9019990	0.0029114	0.0094702	0.1027761	0.5817519	0.0741866	0.0370374	0.5634644
PFI-1	0.0421787	0.6620275	0.0109515	0.9361231	0.8054689	0.2952462	0.0647319	0.4600169	0.1954714	0.1954697	0.8054729	0.3096782	0.0097142	0.1673279	0.4637469
PFI-3	0.5381041	0.2135525	0.0404625	0.0460826	0.3096833	0.5586078	0.0115903	0.0488173	0.0486227	0.0119823	0.0363225	0.4982379	0.1856015	0.0806050	0.8436218
Phthalazinone pyrazole	0.9264181	1.0000000	0.1452191	0.0121036	0.0793083	0.7348866	0.5586037	0.9509067	0.1169567	0.1387971	0.3245839	0.6890077	0.2420876	0.0037010	0.4811078
Piceatannol	0.0604042	0.5865816	0.0771654	0.8777230	0.0421766	0.7348866	0.0097112	0.5179772	0.1973629	0.1571454	1.0000000	0.7348866	0.8776736	0.0095254	0.8436218
Pimelic Diphenylamide 106	0.5900746	0.5419921	0.4363232	0.0876967	0.5380998	0.0741775	0.0904212	0.8898215	0.0853198	0.0046624	0.0363225	0.0488126	0.2812868	0.0121397	0.9326261
Pyroxamide	0.0524526	0.1267097	0.3442411	0.0244372	0.0524502	0.0995576	0.3096732	1.0000000	0.1737953	0.0025681	0.6007483	0.0550604	0.3245839	0.2970985	0.4467339
RG-108	0.8054709	0.3340584	0.7823849	0.4860328	0.0421766	0.2124722	0.3719816	0.7348919	0.5589077	0.0656512	0.4600216	0.6890077	0.6890170	0.4137957	0.6520712
RGFP966	0.3399278	0.4531872	0.3613371	0.1515266	0.0081098	0.3557242	0.0061463	0.3096883	0.0284969	0.0068543	0.1659695	0.9509056	0.4415401	0.0856133	0.0485378
RSC-133	0.2677631	0.5278419	0.2361265	0.5294673	0.0421766	0.2420729	0.7118139	0.7118225	0.9869258	0.1653113	0.8776736	0.6007369	0.7118254	0.0279439	0.0241689
Rucaparib (phosphate)	0.2546978	0.7501869	0.4372365	0.0304738	0.5080529	0.1237461	0.0563039	0.0193047	0.0088853	0.0063112	0.2299186	0.4234767	0.0081112	0.0074254	0.4137957
RVX-208	0.0046238	0.3103248	0.7823849	0.9852442	0.2181815	0.0741775	0.0137864	0.4789236	0.5811601	0.9912836	0.3245839	0.4415258	0.4415401	0.5168881	0.0121397
S-(5'-Adenosyl)-L-methionine (tosylate)	0.9019990	0.7914501	0.2062726	0.6262397	0.5794967	0.8534576	0.4234719	0.1479378	0.5886686	0.7596887	0.0793146	0.1164097	0.3557393	0.1673279	0.6318908
S-Adenosylhomocysteine	0.0055958	0.2828669	0.1195089	0.8099964	0.3399227	0.0933886	0.0050875	0.0262660	0.4220001	0.8143025	0.2299186	0.8054669	0.1755809	0.3671772	0.3379950
SAHA	0.0038072	0.1166750	0.0121318	0.0037820	0.3399227	0.0647346	0.2952411	0.4982468	0.0068545	0.0003186	0.0524551	0.4982379	0.0524551	0.0025506	0.0032910
Salermide	0.4234864	0.6845335	0.2185469	0.5375714	0.3887070	1.0000000	0.5380913	0.4324515	0.3708232	0.0749591	0.2952613	0.4507083	0.7582056	0.6520712	0.7141073
SB939	0.0038072	0.0706339	0.0187721	0.0008153	0.0453936	0.3557242	0.0421723	0.0266602	0.0125509	0.0045056	0.1659695	0.0106146	0.0050893	0.0485378	0.4467339
Scriptaid	0.4600169	0.9354838	0.4146650	0.0412927	0.9509061	0.4415258	0.2952411	0.0209542	0.0873222	0.1359064	0.1094305	0.4789144	0.2181911	0.4467339	0.0080740
SGC-CBP30	0.0647402	0.8459615	0.0289754	0.3780044	0.7233203	0.1755674	0.4600028	0.1567550	0.5086529	0.6346283	0.4789282	0.2181767	0.1164211	0.1114660	0.5384404
SGC0946	0.0055958	0.3968564	0.5921754	0.6262397	0.0421766	0.0074002	0.0246201	0.1149171	0.0416067	0.3791673	0.0046242	0.7181839	0.2677681	0.6931928	0.3978805
SGI-1027	0.0266602	0.5694929	0.1562537	0.0438084	0.5586119	0.7348866	0.3245637	0.7582031	0.5811601	0.9260154	0.4789282	0.7817318	0.1567594	0.2842341	0.0669920
Sinefungin	0.1314465	0.1687892	0.2515055	0.3883058	0.4982424	0.0163409	0.6223225	0.0050888	0.2227416	0.0026148	0.8054729	0.4600075	0.4234913	0.5352780	0.0454129
SIRT1/2 Inhibitor IV	0.0163431	0.9956942	0.4073096	0.7160607	0.6223297	0.0964371	0.5179641	0.1164173	0.8483846	0.1035737	0.3096934	0.7817318	0.7118254	0.2717521	0.2047554
Sirtinol	0.4234864	0.5207548	0.6000100	0.9852442	0.8776710	0.0246216	0.3719816	0.4694129	0.7062496	0.0004091	0.1314505	0.6664780	0.0067491	0.3978805	0.0628986
Sodium 4-Phenylbutyrate	0.6777100	0.2686052	0.5865737	0.0056395	0.0106154	0.3719866	0.1960201	0.1659651	0.7225532	0.0027593	0.9545609	0.2812717	0.1094305	0.0074254	0.0300150
Sodium Butyrate	0.8534606	0.3849375	0.3615907	0.0521443	0.5380998	0.2420729	0.2952411	0.4324515	0.0498836	0.6981456	0.0964475	0.7817251	0.0106171	0.7141073	0.0370374
Splitomicin	0.4789236	0.9784737	0.0223110	0.0947839	0.2677581	0.0563065	0.6007331	0.4694129	0.1169567	0.7931741	0.2299186	0.0363166	0.0067491	0.0224531	0.7673089
Suramin (sodium salt)	0.0563117	0.2503695	0.5732786	0.2293040	0.0266586	0.4324419	0.9754409	0.0964440	0.9869258	0.0208570	0.4982513	0.7118167	0.3024150	0.0121397	0.7565726
Tenovin-1	0.2420827	0.9850393	0.3572015	0.9067547	0.0311688	0.5900669	0.2181719	0.0964440	0.3228227	0.0027593	0.1479420	0.9754412	0.0246261	0.4637469	0.0179254
Tenovin-6	0.2812817	0.9054654	0.3979000	0.8694923	0.7582007	0.3887021	0.6332383	0.0647402	0.0924939	0.0873209	0.6223370	0.9019970	0.0647430	0.2656502	0.0208442
trans-Resveratrol	0.1522936	0.9870832	0.3979000	0.9165320	0.5794967	0.1567464	0.3477538	0.8054709	0.5662791	0.3821344	0.2547028	0.3719866	0.4234913	0.0131428	0.7997862
Trichostatin A	0.0038072	0.0932869	0.0029647	0.0355333	0.9264173	0.7699362	0.1314344	0.0193047	0.6820469	0.3616606	0.1094305	0.2420729	0.0088804	0.0057505	0.0166051
Tubacin	0.0081105	0.1526970	0.3315825	0.2082745	0.3399227	0.1027653	0.8534561	0.0215487	0.9173412	0.0053399	0.2812868	0.6890077	0.5586201	0.3671772	0.0518413
Tubastatin A	0.0050888	0.3233694	0.8473414	0.1654112	0.1755719	0.0041967	0.0106138	0.1314465	0.1772776	0.7721970	0.3887169	0.5794927	0.0604069	0.8216344	0.3103465
Tubastatin A (trifluoroacetate salt)	0.6442521	0.9183334	0.6000100	0.0728159	0.6664813	0.0600521	0.1314344	0.5179772	0.0160021	0.0384322	0.9509072	0.9848459	0.0336632	0.9326261	0.0095254
UNC0224	0.0793114	0.0891508	0.4563076	0.0002473	0.7582007	0.3399177	0.5279745	0.0904313	0.4157133	0.0461802	0.0741866	0.7817318	0.0524551	0.1673279	0.5352780
UNC0321 (trifluoroacetate salt)	0.2299138	0.3286857	0.5536071	0.0947839	0.0421766	0.4789144	0.8534561	0.3719966	0.6900797	0.1195280	0.6664878	0.5586078	0.8054729	0.1051491	0.3103465
UNC0631	0.3887120	0.2593764	0.0029647	0.0008153	0.1094231	0.0741775	0.1479251	0.0106163	0.0220969	0.5086502	0.2299186	0.0227222	0.0055963	0.0052731	0.0131428
UNC0638	0.0038072	0.7257595	0.2138718	0.0008153	0.0604015	0.8534530	0.4146068	0.0115929	0.7062496	0.0172390	0.0847271	0.9264165	0.0097142	0.0074254	0.0057505
UNC0642	0.0038072	0.0813221	0.0619677	0.0008524	0.9264150	0.0193022	0.7935712	0.2314628	0.4742634	0.5886672	0.0288412	0.9754412	0.8534621	0.0396698	0.9102512
UNC0646	0.0038072	0.2415834	0.2682778	0.0008153	0.4415305	0.9264165	0.6777005	0.0209542	0.4157133	0.0000940	0.1479420	0.9141957	0.0067491	0.0396698	0.0370374
UNC1215	0.0506052	0.4796037	0.3512115	0.0796257	0.0488149	0.4415258	0.1960201	0.4140848	0.5601579	0.0043537	0.0453981	0.1960248	0.2299186	0.0424593	0.1506607
UNC1999	0.3557343	0.4276124	0.7314682	0.7057695	0.0693270	0.0041967	0.3557192	0.7467772	0.9433900	0.3391208	0.3399328	0.1094194	0.6664878	0.5730206	0.0087729
Valproic Acid (sodium salt)	0.8414032	0.9651007	0.0328126	0.5664180	0.0150169	0.9019970	0.5380913	0.0793114	0.6740501	0.0146202	0.0453981	0.4982379	0.0170434	0.7997862	0.7352388
WDR5-0103	0.6223334	0.5491660	0.3032357	0.4783478	0.6777068	0.8174017	0.2881918	0.6223334	0.7889672	0.2558890	0.3864183	0.4588341	0.3720016	0.8216344	0.6520712
Zebularine	0.9509067	0.6387161	0.0569979	0.8291661	0.0964406	0.0693241	0.4234719	0.1395047	0.5962216	0.5699801	0.1064317	0.0488126	0.0081112	0.1853437	0.2717521

Compound	J19-2	J19-3	J19-4	J19-5	J19-6	J19-7	J2-2	J21-4	J21-5	J21-7	J4-1	J4-4	J7-6	J8-3	J8-4	J9-3	JEN
(-)-JQ1	0.0193367	0.7141095	0.7889161	0.2047591	0.1853402	0.6725095	0.8455380	0.6031531	0.5168915	0.1211569	0.4600122	0.0532234	0.1960248	0.9509061	0.1094305	0.9428272	0.3481358
(-)-Neplanocin A	0.9550543	0.7352408	0.0806026	0.0279452	0.6119782	0.1211599	0.2383417	0.4784266	0.2047591	0.3379911	0.0964406	0.0242202	0.4234767	0.1395005	0.5817643	0.6240901	0.2962892
(+)-Abscisic Acid	0.0553309	0.0224542	0.1219820	0.2256087	0.0628964	0.3978805	0.7388077	0.6277616	0.2047591	0.0179245	0.1027689	0.8298440	0.2952462	0.4600122	0.0524551	0.2138336	0.0536652
(+)-JQ1	0.0037010	0.3103504	0.0856108	0.0103355	0.3239748	0.0166051	0.0020268	0.0021822	0.0037013	0.4300780	0.5586119	0.0009832	0.4982379	0.0038069	0.0488196	0.0010126	0.2724096
(R)-PFI-2 (hydrochloride)	0.0131428	0.0087734	0.0103343	0.0680763	0.7352367	0.8757279	0.8302744	0.9800599	0.5923569	0.5168847	0.6007407	0.1894841	0.3719866	0.6777068	0.0875401	0.6071771	0.3302474
1-Naphthoic Acid	0.1853437	0.2256087	0.6318881	0.6318936	0.0355046	0.8436218	0.9918191	0.4852737	0.0553329	0.0669898	0.2483244	0.2898592	0.8776698	0.6664813	0.0995682	0.5421340	0.0228739
2-hexyl-4-Pentynoic Acid	0.0153711	0.2479315	0.4637433	0.2349070	0.5730175	0.6725095	0.5436379	0.0940157	0.0590161	0.7565707	0.8293844	0.1763206	0.5380956	0.0453936	0.0266618	0.6666586	0.5492897
2-PCPA (hydrochloride)	0.0166051	0.0057508	0.2842303	0.0792599	0.4988276	0.8436218	0.6768328	0.5452787	0.6119839	0.4467302	0.8776710	0.6235764	0.0163409	0.8054689	0.0604069	0.0271558	0.4955347
2,4-Pyridinedicarboxylic Acid	0.0454129	0.1853473	0.5352747	0.0131435	0.0690121	0.3103465	0.8777666	0.2576597	0.1761676	0.7141051	0.8776710	0.3213965	0.7348866	0.5586119	0.8534621	0.3764218	0.2647508
2',3',5'-triacetyl-5-Azacytidine	0.3379950	0.6828214	0.0424576	0.4637505	0.0396682	0.8216344	0.1942874	0.7376877	0.7780954	0.8436205	0.6223297	0.2662305	0.3972237	0.6664813	0.8174073	0.2603867	0.4141359
3-amino Benzamide	0.9326261	0.1853473	0.2970947	0.6725120	0.3103426	0.2047554	0.3614776	0.9163965	0.5923569	0.0224520	0.0311688	0.7854615	0.1027653	0.4234816	0.4694176	0.2767283	0.1962694
3-Deazaneplanocin A	0.0112051	0.0037013	0.3239748	0.4137995	0.5539888	0.0582221	0.0908406	0.0506403	0.6725120	0.3978767	0.2546929	0.0943892	0.2677531	0.6622140	0.5381083	0.2603867	0.0682555
3-Deazaneplanocin A (hydrochloride)	0.0224531	0.0518432	0.2717483	0.5730237	0.3523910	0.7352388	0.9754608	0.2692783	0.0103355	0.0669898	0.2812767	0.0037958	0.9509056	0.7348893	0.7582056	0.2767283	0.3087383
3,3'-Diindolylmethane	0.0908702	0.6520738	0.3823360	0.6894124	0.6931904	0.2479277	0.0467367	0.3247440	0.3171145	0.5168847	0.3399227	0.7714595	0.3719866	0.9264173	0.8054729	0.8785224	0.8734176
4-iodo-SAHA	0.3379950	0.2717560	0.0062661	0.3239825	0.7141051	0.5260387	0.1143287	0.3346646	0.0590161	0.7565707	0.2181815	0.0035605	0.1027653	0.2420778	0.0904347	0.0039691	0.1673515
5-Azacytidine	0.9102512	0.8216358	0.6931904	0.2842380	0.4637433	0.3823399	0.3507890	0.8387906	0.1352932	0.6318881	0.6664813	0.9122691	0.3245586	0.6553234	0.0363225	0.2273453	0.5771057
5-Methyl-2'-deoxycytidine	0.0153711	0.0087734	0.4637433	0.3219574	0.4137919	0.5539920	0.8957811	0.6248123	0.0629007	0.3671733	0.3719916	0.3830268	0.9264165	0.3887070	0.4058817	0.0646014	0.2647508
5-Methylcytidine	0.0208442	0.2047591	0.4988276	0.4988345	0.8436205	1.0000000	0.8302744	0.0030341	0.0166060	0.9775181	0.6332454	0.1409226	0.4600075	0.3557293	0.4058817	0.1962827	0.4154750
6-Thioguanine	0.0037010	0.0131435	0.0485360	0.4300854	0.1211569	0.0241689	0.2529564	0.0257840	0.0037013	0.9550540	0.8293844	0.0008519	0.9019970	0.1314425	0.0904347	0.0687260	0.0044164
a-Hydroxyglutaric Acid (sodium salt)	0.0628986	0.9214309	0.0193357	0.3978843	0.7458774	0.7780936	0.0229374	0.8768516	0.5730237	0.5168847	0.7699385	0.5280319	0.3096782	0.3887070	0.9020000	0.4959699	0.0976873
AGK2	0.4137957	0.2365840	0.0153702	0.1376057	0.0179245	0.6931928	0.2199917	0.4443845	0.0193377	0.7565707	0.4234816	0.2179572	0.1094194	1.0000000	0.5882788	0.1689505	0.0573794
AGK7	0.0121397	0.8657328	0.0628964	0.1506640	0.8216330	0.5730206	0.0649242	0.9081346	0.0322180	0.4300780	0.0793083	0.2332950	0.0711631	0.4600122	0.0904347	0.0534367	0.1324067
AK-7	0.0396698	0.0037013	0.1352869	0.1376057	0.2149944	0.3239787	0.8210017	0.9163965	0.7141095	0.0131420	0.0524502	0.1700002	0.7817318	0.6664813	0.0177711	0.6666586	0.6838990
Anacardic Acid	0.2047554	0.4057915	0.0370359	0.2159737	0.2365765	0.4637469	0.1304453	0.9005479	0.5445950	0.2149944	0.5586119	0.5021711	0.2812717	0.3399227	0.1395088	0.7075421	0.4684204
Apicidin	0.7565726	0.0037013	0.0037007	0.0040482	0.0037007	0.2479277	0.0013305	0.1176739	0.0048321	0.3823360	0.1237500	0.0007925	0.4415258	0.0067480	0.4694176	0.1118995	0.0039086
BIX01294 (hydrochloride hydrate)	0.0179254	0.2479315	0.1761607	0.2762294	0.7141051	0.7997862	0.9715018	0.9561493	0.0485396	0.7997846	0.9754414	0.0007925	0.0106146	0.1237500	0.5795046	0.1040156	0.3439523
BML-210	0.0300150	0.0142192	0.1853402	0.7997878	0.3103426	0.2970985	0.0583030	0.4809190	0.4988345	0.0142177	0.3245738	0.1068338	0.2420729	0.8054689	0.6007483	0.6071771	0.1902108
BRD73954	0.7141073	0.0758397	0.3523910	0.8436230	0.5352747	0.3239787	0.0441678	0.1350112	0.6520738	0.5539888	0.3170662	0.3515972	0.1094194	0.0126476	0.0097142	0.1777246	0.8129704
Bromosporine	0.0454129	0.4057915	0.6725070	0.8484173	0.5352747	0.7780936	0.0035282	0.2132452	0.0044244	0.7565707	0.2181815	0.0094939	0.9754412	0.0038069	0.0209555	0.3376896	0.2391188
BSI-201	0.0553309	0.0590161	0.2047518	0.6520738	0.5539888	0.4988310	0.8777666	0.0393443	0.1021641	0.1506574	0.0453936	0.2495350	0.0209515	0.4234816	0.0488196	0.5906649	0.3666282
Butyrolactone 3	0.9438342	0.8216358	0.5168847	0.1024179	0.0485360	0.4988310	0.0702859	0.2024211	0.1761676	0.1211569	0.5948413	0.3835362	0.0453913	1.0000000	0.0600605	0.9889139	0.8188968
C646	0.0068344	0.1351806	0.1511692	0.0150414	0.4521211	0.1940474	0.7819014	0.4862988	0.3435550	0.0309276	0.3096833	0.5073478	0.2546879	0.5586119	0.0081112	0.8953956	0.0132012
CAY10398	0.7565726	0.0037013	0.2256012	0.0424609	0.0166043	0.5923539	0.0978743	0.0034526	0.1352932	0.0908624	0.3096833	0.0009154	0.1567464	0.0038069	0.0097142	0.2060773	0.0926271
CAY10591	0.0300150	0.0087734	0.5168847	0.0131435	0.0963808	0.0300150	0.5436379	0.3296792	0.4988345	0.5539888	0.2952512	0.8964010	0.9754412	0.0741806	0.0647430	0.0358952	0.3045514
CAY10603	0.4467339	0.0040482	0.0087724	0.3222502	0.0103343	0.7352388	0.5920474	0.3371745	0.2971024	0.8436205	0.3887070	0.0027462	0.1479294	0.0106154	0.8534621	0.0730645	0.2684420
CAY10669	0.5260387	0.2479315	0.1021586	0.1352932	0.1145389	0.8216344	0.8535724	0.6883289	0.4137995	0.2365765	0.6223297	0.6886815	0.1394964	0.1707112	0.2952613	0.2009175	0.3527014
CAY10683	0.0590141	0.5260420	0.9326256	0.0420510	0.2365765	0.2656502	0.5920474	0.1661575	0.2479315	0.2047518	0.3096833	0.1578712	0.5794927	0.4058719	0.1027761	0.6666586	0.0400686
CBHA	0.0553309	0.0259984	0.3239748	0.0298538	0.0629868	0.1352900	0.7298393	0.9481868	0.4637505	0.0856108	0.2181815	0.0668110	0.2124722	0.2420778	0.9754420	0.1370829	0.0040108
CCG-100602	0.2842341	0.1280766	0.8436205	0.0590161	0.5923509	0.9775183	0.6445093	0.2693917	0.0629007	0.5352747	0.1395005	0.0962628	0.6777037	0.6890108	0.3096934	0.9713954	0.3045514
Chidamide	0.0142184	0.0037013	0.5352747	0.0355077	0.2717483	0.4141066	0.1708589	0.6929152	0.1428199	0.1948687	0.1960294	0.7259163	0.9019970	0.0137874	0.2812868	0.5501898	0.9219406
CI-994	0.2365802	0.0501668	0.3239748	0.2256087	0.5539888	0.3978805	0.1119976	0.5519421	0.3671810	0.0908676	0.5179728	0.1894841	0.0081092	0.0074009	0.0097142	0.8203446	0.1842912
Cl-Amidine (hydrochloride)	0.2149981	0.1350800	0.0628964	0.0241700	0.0241677	0.2596508	0.3507890	0.0706368	0.8216358	0.9102505	0.9019980	0.2898592	0.4415258	0.2181815	0.6007483	0.0710963	0.3954914
coumarin-SAHA	0.1428167	0.0037013	0.5168847	0.0987919	0.0052727	0.0062665	0.0552089	0.0542891	0.0080745	0.1428135	0.9509061	0.0009832	0.0150158	0.7582007	0.4789282	0.2846650	0.0778971
CPI-203	0.0037010	0.0080745	0.4637433	0.6119839	0.3379911	0.5539920	0.0063915	0.0040494	0.0037013	0.1428071	0.0741806	0.0012156	0.0266570	0.0038069	0.0041979	0.0067388	0.0067404
CPTH2 (hydrochloride)	0.0208442	0.1145447	0.4137919	0.7565745	0.0396682	0.1853437	0.6594987	0.0665731	0.0208452	0.0166043	0.9754414	0.0729923	0.1200301	0.0097127	0.0046242	0.7492867	0.3028521
CUDC-101	0.4137957	0.0037013	0.0345538	0.0345567	0.3239748	0.0420492	0.0035282	0.4809190	0.0279452	0.8216330	0.0904279	0.7712214	0.0038065	0.1237500	0.9020000	0.4614729	0.0558984
Daminozide	0.2596508	0.0553329	0.6318881	0.0095260	0.3823360	0.9550543	0.6592879	0.6404554	0.1280766	0.2842303	0.1164135	0.7712214	0.1394964	0.0061474	0.0693329	0.9333195	0.3760999
Decitabine	0.0068344	0.0072473	0.1511692	0.7621169	0.3742237	0.6745731	0.0615400	0.8111082	0.6889003	0.4759543	0.3557293	0.0831344	0.4982379	0.7582007	0.4058817	0.6465732	0.0563868
Delphinidin (chloride)	1.0000000	0.0166060	0.0300137	0.0806075	0.9550540	0.7352388	0.7819014	0.9780815	0.3523988	0.2149944	0.5794967	0.3312695	0.9386532	0.0904279	0.6890170	0.3211602	0.7299503
DMOG	0.1428167	0.7141095	0.1588256	0.8484173	0.0713001	0.1853437	0.0522528	0.6672729	0.4811184	0.9326256	0.4415305	0.1520580	0.2068714	0.3557293	0.0693329	0.6071771	0.6415309
Ellagic Acid	0.1021613	0.1673313	0.9326256	0.0279452	0.0095248	0.5730206	0.5124974	0.2441359	0.0044244	0.3523910	0.4324467	0.1998201	0.8054669	0.9019980	0.6442555	0.9225171	0.1816731
EPZ005687	0.3103465	0.0224542	0.5730175	0.4762021	0.3978767	0.0590141	0.8957826	0.3447775	0.4300854	0.1021586	0.6007407	0.6739907	0.6007369	0.0693270	0.4415401	0.8348996	0.7543437
EPZ5676	0.0553309	0.9662830	0.0495830	0.2150018	0.6931904	0.3379950	0.0091098	0.8259135	0.0131435	0.9102505	0.3096833	0.3022331	0.3802829	0.9509061	0.0115938	0.5878897	0.0191262
Etoposide	0.1352900	0.0629007	0.3523910	0.0121404	0.0080735	0.4300817	0.6086126	0.5255311	0.0224542	0.3103426	0.0453936	0.1795438	0.8293827	0.0126476	0.0964475	0.2603867	0.5364423
EX-527	0.1021613	0.0068243	0.0806026	0.0057508	0.5539888	0.3239787	0.0721538	0.2628316	0.1588323	0.2256012	0.6442486	0.4287861	0.7118167	0.6442486	0.1314505	0.5501898	0.0294538
F-Amidine (trifluoroacetate salt)	0.1211599	0.5352813	0.0424576	0.2717560	0.3239748	0.6520712	0.6892428	0.3530812	0.5352813	0.4300780	0.9754414	0.1010081	0.6664780	0.5586119	0.3399328	0.5580881	0.2219175
Garcinol	0.5923539	0.3978843	0.1853402	0.5168915	0.1948687	0.3379950	0.3834596	0.8259135	0.3597384	0.1280705	0.0847206	0.8964010	0.0603988	0.2611640	0.5586201	0.1066102	0.4899540
Gemcitabine	0.0037010	0.0037013	0.0044238	0.0037013	0.0040476	0.0037010	0.0051233	0.0001960	0.0037013	0.3978767	0.2546929	0.0008519	0.0299804	0.0038069	0.0038076	0.0019631	0.3481358
GSK-J1 (sodium salt)	0.3978805	0.3103504	0.3103426	0.0224542	0.3379911	0.4988310	0.6742008	0.7987719	0.2047591	0.4300780	0.6890108	0.4525286	0.4982379	0.8293844	0.1314505	0.1415115	0.3573066
GSK-J2 (sodium salt)	0.7352388	0.0068243	0.7565707	0.0087734	0.0908676	0.7780936	0.0578438	0.5202908	0.1280766	0.0193357	0.2181815	0.9600583	0.5279788	0.1522893	0.0088804	0.1730019	0.3573066
GSK-J4 (hydrochloride)	0.5923539	0.8106918	0.1280705	0.2596546	0.6931904	0.9031885	0.1074448	0.4747186	0.6931951	0.0396682	0.1314425	0.9920085	0.6223261	0.9754414	0.0717231	0.0224321	0.5011509
GSK-LSD1 (hydrochloride)	0.0103349	0.0062669	0.5923509	0.2150018	0.4300780	0.1547006	0.1079197	0.1042477	0.0713046	0.8216330	0.6007407	0.0297435	0.0453913	0.0041971	0.0847271	0.0606833	0.8111220
GSK126	0.1280735	0.8216358	0.3671733	0.1698394	0.2047518	0.6318908	0.3229641	0.9299421	0.5539952	0.5539888	0.8293844	0.4287861	0.9509056	0.7348893	0.5179815	0.7704468	0.0016212
GSK2801	0.0485378	0.2256087	0.3823360	0.1981648	0.4300780	0.5901168	0.9335671	0.1130556	0.1948759	0.6931904	0.7118196	0.2309155	0.0115912	0.1027689	0.8776736	0.6465732	0.7769977
GSK343	0.3671772	0.0057508	0.3171068	0.0629007	0.1145389	0.7352388	0.7841693	0.7301587	0.9326266	0.9102505	0.9264173	0.1046723	0.3887021	0.0055963	0.0604069	0.8675756	0.9435502
GSK4112	0.3239787	0.0713046	0.7997846	0.0485396	0.1673245	0.5168881	1.0000000	0.6709135	0.7780954	0.0193357	0.6442486	0.2033580	0.5690005	0.8534591	0.0088804	0.4659304	0.4256865
HC Toxin	0.0037010	0.0037013	0.0057501	0.0040482	0.0037007	0.0037010	0.0028475	0.0006398	0.0037013	0.0518394	0.3887070	0.0007925	0.0038065	0.0038069	0.7348946	0.0008536	0.0181455
HNHA	0.0518413	0.1900640	0.5352747	0.8045514	0.0908676	0.6218986	0.8487516	0.3550842	0.1428199	1.0000000	0.7817340	0.7714575	0.3096782	0.7817340	0.0904347	0.4785516	0.2554586
HPOB	0.0259972	0.0166060	0.6931904	0.1809070	0.5168847	0.2842341	0.9525210	0.7414615	0.0241700	0.2256012	0.9509061	0.1004565	0.3399177	0.9754414	0.0227265	0.7283136	0.2647508
I-BET151	0.0908702	0.0454146	0.0396682	0.6520738	0.5352747	0.2842341	0.0030442	0.4747186	0.0057508	0.2365765	0.1960294	0.0011330	0.0061468	0.0038069	0.0041979	0.1692667	0.0781770
I-BET762	0.0000650	0.6745709	0.1403543	0.3433575	0.9922090	0.8071408	0.0070042	0.0000349	0.0000551	0.3955860	0.3096833	0.0021055	0.0246216	0.0038069	0.0227265	0.0042807	0.2962892
I-CBP112 (hydrochloride)	0.2717521	0.4467375	0.4811113	0.2159737	0.2717483	0.2596508	0.0666718	0.3871565	0.1352932	0.1506574	0.7817340	0.2577112	0.1027653	0.7582007	0.1755809	0.2266968	0.0447233
IOX1	0.8768264	0.6119839	0.0166043	0.0121404	0.5730175	0.8657317	0.0423378	0.6326133	0.5352813	0.3671733	0.2546929	0.1464093	0.7348866	0.5179728	0.0336632	0.2611977	0.3391159
Isoliquiritigenin	0.1466954	0.1588323	0.0758350	0.7997878	0.5168847	0.2479277	0.9591127	0.0911078	0.2150018	0.3671733	0.1164135	0.0166729	0.1855877	0.6890108	0.6223370	0.6240901	0.4679698
ITF 2357	0.5923539	0.0037013	0.3823360	0.6931951	0.3103426	0.8879473	0.0051233	0.6242195	0.1588323	0.6622538	0.6007407	0.0019683	0.6664780	0.8054689	0.4789282	0.6072748	0.0046209
JGB1741	0.5168881	0.0259984	0.3239748	0.0856158	0.1428135	0.0590141	0.5727968	0.4624505	1.0000000	0.9775181	0.5794967	0.0149368	0.2239807	0.4600122	0.4415401	0.0433822	0.4955347
JIB-04	0.0037010	0.0037013	0.0037007	0.0074258	0.0037007	0.0037010	0.4389092	0.0010978	0.0080745	0.6931904	0.3887070	0.3413365	0.0061468	0.0097127	0.0038076	0.1777246	0.3857212
LAQ824	0.0037010	0.0037013	0.0037007	0.0037013	0.0037007	0.0037010	0.0035282	0.0000801	0.0037013	0.1761607	0.8293844	0.0007925	0.8776698	0.0038069	0.0038076	0.0036842	0.2321283
Lestaurtinib	0.0037010	0.0037013	0.0087724	0.0355077	0.0037007	0.0370374	0.0035282	0.0058815	0.0037013	0.0142177	0.9509061	0.0007925	0.7118167	0.0227236	0.5279917	0.0982333	0.8188968
Lomeguatrib	0.8879473	0.0057508	0.3379911	0.5352813	0.3823360	0.0963835	0.3229641	0.7910601	0.8879482	0.0142177	0.8414016	0.9920085	0.7118167	0.3399227	0.0288412	0.3096380	0.4465433
M 344	0.0856133	0.0040482	0.1352869	0.0241700	0.0142177	0.1588289	0.0954819	0.5452787	0.0103355	0.7565707	0.6442382	0.0009154	0.2546879	0.0847206	0.3096934	0.0047928	0.0371994
MC 1568	0.1428167	0.3978843	1.0000000	0.2047591	0.0553289	0.2596508	0.8025306	0.3982278	0.6318936	0.3103309	0.6890108	0.7259163	0.0563065	0.4058719	0.4885337	0.4785516	0.0058657
Methylstat (hydrate)	0.9550543	0.6931951	0.9775181	0.1280766	0.3379911	0.7565726	0.8616235	0.8387906	0.0629007	0.0758350	0.7118196	0.0831344	0.0563065	0.8776710	0.0604069	0.0622320	0.3964914
MI-2 (hydrochloride)	0.6520712	0.7780954	0.7352367	0.0279452	0.2717483	0.6622563	0.4541594	0.0810443	0.9326266	0.2479239	0.9019980	0.9043299	0.1164097	0.7817340	0.0266618	0.8577093	0.8456097
MI-nc (hydrochloride)	0.8436218	0.0048321	0.5078106	0.7246468	0.3671733	0.2842341	0.3114417	0.1108007	0.3823437	0.0166043	0.4234816	0.0013977	0.0311670	0.0209528	0.0115938	0.3813718	0.0636526
Mirin	0.2149981	0.0758397	0.3523910	0.6921510	0.0179245	0.3036706	0.3288259	0.1350112	0.1506640	0.5826438	0.8534591	0.4585762	0.7118167	0.5794967	0.6890170	0.3104304	0.1718920
MS-275	0.0300150	0.0080745	0.5445886	0.1024179	0.6520685	0.2149981	0.1862294	0.4809190	0.0095260	0.0856108	0.8174035	0.1025486	0.5586078	0.3096833	0.2420876	0.2375801	0.1767321
N-Oxalylglycine	0.0908702	0.0322180	0.4467302	0.0249918	0.4467302	0.7565726	0.5436379	0.7226550	0.0908728	0.1761607	0.6890108	0.9122691	0.2420729	0.9264173	0.1164471	0.1868335	0.2134864
Nicotinamide	0.0590141	0.1853473	0.5730175	0.3171145	0.4988276	0.1506607	1.0000000	0.6795727	0.1021641	0.9326256	0.1960294	0.7358892	0.1237461	0.6664813	0.5179815	0.1926269	0.3964914
Octyl-a-ketoglutarate	0.2717521	0.1506640	0.5352747	0.3978843	0.0310964	0.9102512	0.8858571	0.8454308	0.1761676	0.1716966	0.1659607	0.5215018	0.3170611	0.6442486	0.0115938	0.6756945	0.3760999
OTX015	0.0087729	0.4137995	0.2173032	0.8657328	0.0424576	0.3379950	0.0229374	0.0109475	0.0037013	0.0179245	0.0209528	0.0126311	0.5380956	0.0038069	0.0693329	0.0056912	0.4899540
Oxamflatin	0.0963835	0.0040482	0.4467302	0.5901199	0.0095248	0.4988310	0.1942874	0.4443836	0.0629007	0.6318881	0.0904279	0.0012156	0.2812717	0.0904279	0.0055963	0.3966708	0.0516318
Panobinostat	0.0037010	0.0037013	0.0037007	0.0037013	0.0037007	0.0037010	0.0035282	0.0000504	0.0037013	0.0037007	1.0000000	0.0007925	0.7817318	0.0038069	0.3399328	0.0036842	0.2024635
PCI 34051	0.3239787	0.0908728	0.0357750	0.2047591	0.3379911	0.7780884	1.0000000	0.7987719	0.9550547	0.2421997	0.0170411	0.8178043	0.3557242	0.3887070	0.6890170	0.6756945	0.0494219
PFI-1	0.4467339	0.1761676	0.3671733	0.0485396	0.8436205	0.6725095	0.0346683	0.1199360	0.0571478	0.8216330	0.0964406	0.0905092	0.0299804	0.4058719	0.0038076	0.1415115	0.3215304
PFI-3	0.0259972	0.0179263	0.0806026	0.0273296	0.2256012	0.8879473	0.1244980	0.9163965	0.0396714	0.0628964	0.4415305	0.4894966	1.0000000	0.4058719	0.3245839	0.4283590	0.3436059
Phthalazinone pyrazole	0.4637469	0.0345567	0.4300780	0.5352813	0.3823360	0.5445918	0.5183023	0.1515279	0.1673313	0.0289609	0.7118196	0.4358124	0.0177675	0.5179728	0.4415401	0.0278553	0.0138708
Piceatannol	0.2479277	0.3671810	0.7352367	0.1948759	0.0590121	0.2443368	0.1942874	0.9641162	0.1178144	0.1352869	0.0363186	0.7868262	0.2068856	0.0177687	0.2677681	0.0982333	0.7500011
Pimelic Diphenylamide 106	0.0669920	0.2479315	0.1506574	0.5199654	0.9550540	0.7780936	0.2629775	0.4594109	0.3523988	0.3523910	0.3557293	0.1464093	0.2546879	0.6442486	0.8534621	0.5878897	0.0046328
Pyroxamide	0.1948723	0.0142192	0.8436205	0.1658135	0.2047518	0.8436218	0.1079197	0.5125723	0.1506640	0.0713001	0.8293844	0.0021055	0.1567464	0.0193035	0.3927149	0.0454070	0.1387040
RG-108	0.1352900	0.9438347	0.2256012	0.3671810	0.0806026	0.0669920	0.8769732	0.8065035	0.0908728	0.4988276	0.7817340	0.4057845	0.8534576	0.3096833	0.4415401	0.1646840	0.7952552
RGFP966	0.0142184	0.0279452	0.5730175	0.6318936	0.4811113	0.0758374	0.6943399	0.5386554	0.0279452	0.7997846	0.7582007	0.3022331	0.8534576	0.5794967	0.4600216	0.4283590	0.2219175
RSC-133	0.0518413	0.0040482	0.3036667	0.6318936	0.6318881	0.0963835	0.2111656	0.8026351	0.1761676	0.0424576	0.0363186	0.1254088	0.6664780	0.5586119	0.6664878	0.4699659	0.0168583
Rucaparib (phosphate)	0.0153711	0.0241700	0.8216330	0.0068243	0.3671733	0.9550543	0.3668954	0.2507938	0.2842380	0.1211569	0.8054689	0.1464093	0.0847174	0.9019980	0.0050893	0.7469045	0.3045514
RVX-208	0.0279439	0.0103355	0.5730175	0.7943678	0.0553289	0.1428167	0.2384312	0.0081849	0.5539952	0.3239748	0.5080529	0.1459773	0.2812717	0.0421766	0.1314505	0.7492867	0.8042664
S-(5'-Adenosyl)-L-methionine (tosylate)	1.0000000	0.0992389	0.4988276	0.2596546	0.4723798	0.3379950	0.0885905	0.4038333	0.6318936	0.3823360	0.6442486	0.6165074	0.8054669	0.2952512	0.9509072	0.1131897	0.0728852
S-Adenosylhomocysteine	0.0495849	0.0052734	0.8879464	0.7412883	0.0454111	0.1428167	0.2480492	0.0727246	0.4137995	0.8879464	0.1479336	0.5073478	0.8776698	0.0453936	0.9754420	0.9446124	1.0000000
SAHA	0.0176533	0.0000551	0.0266499	0.3114625	0.0543985	0.8375271	0.1366107	0.0241408	0.0340977	0.0874832	0.7817340	0.0007925	0.0126467	0.5307924	0.3557393	0.9889139	0.2088082
Salermide	0.3379950	0.1113411	0.3823360	0.0806075	0.0370359	0.4300817	0.5048581	0.3576915	0.0908728	0.2842303	0.1755719	1.0000000	0.0288362	0.8293844	0.0050893	0.1822334	0.2368175
SB939	0.4137957	0.0037013	0.0037007	0.1948759	0.0087724	0.7780936	0.0023248	0.4151880	0.0037013	0.0259960	0.4982424	0.0007925	0.0081092	0.5586119	0.2181911	0.0015377	0.0039086
Scriptaid	0.2596508	0.0037013	0.2479239	0.1761676	0.0259960	0.3379950	0.0987797	0.0047384	0.3309388	0.5539888	0.6890108	0.9920085	0.2299041	0.1164135	0.0524551	0.0023041	0.0046209
SGC-CBP30	0.7920530	0.7695684	0.4289776	0.1552214	0.5004603	0.6745731	0.5124974	0.9365033	0.4065447	0.6462759	0.4789190	0.5021711	0.7581982	0.1479336	0.0311723	0.8785224	0.2554605
SGC0946	0.0279439	0.0044244	0.0241677	0.0987919	0.0518394	0.3392243	1.0000000	0.0050425	0.5923569	0.8216330	1.0000000	0.7184252	0.4600075	0.0209528	0.2952613	0.5137209	0.2498817
SGI-1027	0.5352780	0.2906185	0.5352747	0.1761676	0.0518394	0.5923539	0.7976920	0.1335901	0.9102519	0.6143188	0.8655479	0.5085710	0.2677531	0.7582007	0.0363225	0.2366823	0.0796865
Sinefungin	0.0142184	0.0037013	0.0345538	0.5352813	0.8879464	0.4988310	0.6253759	0.3054831	0.6931951	0.4300780	0.4058719	0.5885966	0.0115912	0.2624425	0.1479420	0.9889139	0.2498817
SIRT1/2 Inhibitor IV	0.2479277	0.0713046	0.7780919	0.3222502	0.3671733	0.3671772	0.3591764	0.2169445	0.1051519	0.5168847	0.4789190	0.1963313	0.0793052	0.2611640	0.0074021	0.5317969	0.0778971
Sirtinol	0.4300817	0.4637505	0.0300137	0.8657328	0.1506574	0.2365802	0.5589215	0.3852920	1.0000000	0.2098242	0.0847206	0.9600583	1.0000000	0.3321859	0.8534621	0.8577093	0.0943564
Sodium 4-Phenylbutyrate	0.3379950	0.5730237	0.2256012	0.0518432	0.1673245	0.9326261	0.0679837	0.6326126	0.0485396	0.4137919	0.4600122	0.2179572	0.4058670	0.3887070	0.4234913	0.3960796	0.2724096
Sodium Butyrate	0.0669920	0.7889195	0.4467302	0.0345567	0.0628964	0.0669920	0.6594987	0.5125723	0.0095260	0.1021586	0.4415305	0.3620512	0.1164097	0.0177652	0.6007483	0.5878897	0.8726648
Splitomicin	0.1021613	0.1428199	0.0469499	0.0987919	0.0166043	0.5168881	0.8302744	0.5978422	0.0553329	0.7565707	0.6223297	0.7714595	0.7233175	0.0904279	0.7757197	0.8785224	0.2957454
Suramin (sodium salt)	0.0758374	0.9326266	0.8436205	0.0669942	0.5539888	0.1506607	0.6253810	0.6722199	0.0669942	0.5352747	0.2812767	0.0544680	0.0391526	0.3637986	0.4058817	0.6156546	0.3391159
Tenovin-1	0.1506607	0.1245793	0.4137919	0.1280766	0.3239748	0.0518413	0.6086126	0.4038343	0.0963862	0.2479239	0.8049702	1.0000000	0.0363166	0.0904279	0.8293879	0.0848945	0.5165127
Tenovin-6	0.0806050	0.1506640	0.9775181	0.0396714	0.1948687	0.0713023	0.0721538	0.2915450	0.1428199	0.0590121	0.0193035	0.8100302	0.4789144	0.0604015	0.2547028	0.9004815	0.1567263
trans-Resveratrol	0.1082115	0.0370389	1.0000000	0.8879482	0.7141051	0.1853437	0.1635395	0.2302544	0.9031894	0.4637433	0.3719916	0.6666927	0.7699362	0.8655479	0.3024150	0.9004815	0.1718920
Trichostatin A	0.3523949	0.0037013	0.0485360	0.0142192	0.0068234	0.7780936	0.0035282	0.0727246	0.0087734	0.0424576	0.9264173	0.0007925	0.1314384	0.0246231	0.0088804	0.1605055	0.0136946
Tubacin	0.1588289	0.0087734	0.2256012	0.5352813	0.3978767	0.9307655	0.1708589	0.9005481	0.1352932	0.6318881	0.7582007	0.8334041	0.3245687	0.4058719	0.3720016	0.2723407	0.6326810
Tubastatin A	0.0345553	0.0259984	0.2256012	0.2047591	0.0259960	0.3823399	0.5436379	0.0267978	0.7141095	0.3379911	0.2546929	0.7358892	0.3245687	0.0050884	0.4234913	0.7283136	0.6076988
Tubastatin A (trifluoroacetate salt)	0.4811149	0.4467375	0.1948687	0.4549569	0.6520685	0.5923539	0.2508089	0.4809190	0.3103504	0.6520685	0.2952512	1.0000000	0.4982379	0.0741806	0.0227265	0.1415115	0.0827752
UNC0224	0.0518413	0.0037013	0.1588256	0.1592874	0.7565707	0.7412862	0.9335671	0.7872115	0.3978843	0.6520685	0.6664813	0.3581702	0.4600075	0.8054689	0.0038076	0.5137209	0.0682555
UNC0321 (trifluoroacetate salt)	0.4057877	0.8216358	0.2047518	0.2047591	0.1924939	0.0590141	0.6253810	0.3499078	0.1673313	0.3103426	0.0563091	0.4769963	0.8655465	0.3719916	0.0137894	0.8577093	0.3760999
UNC0631	0.9584259	0.0037013	0.0259960	0.2762294	0.0289609	0.6021285	0.0156329	0.0622462	0.0908728	0.3523910	0.4789190	0.0007925	0.0046229	0.0137874	0.1094305	0.0128765	0.0976873
UNC0638	0.0518413	0.0112057	0.6520685	0.0424609	0.2047518	0.2596508	0.0695465	0.5565667	0.2971024	0.0193357	0.8534591	0.0007925	0.0038065	0.3399227	0.0055963	0.2983922	0.3760999
UNC0642	0.0370374	0.0068243	1.0000000	0.5923569	0.3103426	0.0908702	0.2384312	0.4123315	0.1390142	0.5539888	0.7582007	0.0185836	0.3477588	0.0193035	0.1094305	1.0000000	0.0176161
UNC0646	0.5730206	0.0037013	0.2479239	0.5539952	0.0485360	0.2365802	0.2862612	0.6563995	0.0908728	0.5352747	0.2420778	0.0018394	0.0038065	0.3096833	0.0288412	0.0142734	0.0036944
UNC1215	0.0279439	0.0037013	0.0424576	0.1658135	0.1853402	0.5901168	0.4972833	0.4443845	0.4300854	0.4637433	0.7817340	0.0484764	0.9631687	0.2812767	0.0227265	0.8348996	0.9154137
UNC1999	0.0179254	0.0424609	0.0300137	0.3945284	0.1145389	0.3671772	0.2946702	0.0068442	0.8216358	0.3671733	0.6890108	0.7534908	0.8054669	0.0055953	0.1314505	0.8566563	0.4360430
Valproic Acid (sodium salt)	0.5730206	0.4637505	0.1506574	0.0582242	0.0609265	0.4300817	0.8863694	0.1764604	0.3523988	0.6318881	0.2169794	0.7109392	0.7348866	0.7118196	0.3720016	0.5783505	0.3478586
WDR5-0103	0.1021613	0.0232973	0.1506574	0.4342563	0.7780919	0.3978805	0.6768328	0.2736356	0.3103504	0.8436205	0.7118196	0.9760286	0.8293827	0.2068904	0.0289443	0.2973612	0.6306418
Zebularine	0.0495849	0.2256087	0.8546602	0.0553329	0.3523910	1.0000000	0.8939606	0.7068744	0.1547039	0.9438338	0.8776710	0.1464093	0.4982379	0.3245738	0.1479420	0.7833833	0.3215304

Supplementary Table S3. Average cell viability for each epigenetic compound. Table of the 139 epigenetic compounds screened, ranked by broad effectiveness. Average cell viability is reported as a percentage normalized to the untreated tumor control cohort and reported with standard deviation. p values are calculated with the Student's t test (confidence level of 0.95) between the group treated with the epigenetic compound and the untreated control group.

Compound	Avg. Cell Viability	Std. Deviation	p Value
Panobinostat	51.9%	23.9	1.06E-40
LAQ824	56.1%	24.0	2.16E-36
HC Toxin	59.4%	24.7	3.16E-32
Gemcitabine	71.4%	22.3	6.58E-25
JIB-04	77.3%	28.9	4.60E-13
SB939	79.6%	23.7	9.30E-15
Apicidin	80.1%	22.9	4.95E-15
UNC0631	81.5%	27.7	4.51E-10
Trichostatin A	81.7%	21.3	8.22E-15
6-Thioguanine	82.8%	21.0	8.78E-14
CPI-203	83.1%	20.6	7.14E-14
UNC0646	83.4%	26.5	2.03E-09
CAY10398	83.5%	20.3	1.77E-13
(+)-JQ1	84.3%	19.5	2.13E-13
Lestaurtinib	84.6%	18.3	5.49E-14
MI-nc (hydrochloride)	85.8%	21.2	2.53E-10
UNC0638	86.4%	26.9	7.58E-07
CAY10603	86.6%	19.7	2.49E-10
BIX01294 (hydrochloride hydrate)	86.7%	26.2	9.70E-07
I-BET762	87.3%	16.9	1.05E-15
M 344	88.1%	18.1	6.11E-10
4-iodo-SAHA	88.1%	18.9	2.75E-09
OTX015	88.8%	18.7	1.21E-08
SAHA	89.1%	19.6	6.53E-10
Oxamflatin	89.6%	16.0	1.23E-09
I-BET151	90.4%	15.8	8.46E-09
coumarin-SAHA	90.6%	15.8	1.74E-08
ITF 2357	91.5%	18.6	7.91E-06
CUDC-101	92.1%	17.4	1.41E-05
UNC0642	92.4%	22.1	6.10E-04
MS-275	92.5%	17.3	2.19E-05
CPTH2 (hydrochloride)	92.6%	12.8	4.86E-08
3-Deazaneplanocin A	92.8%	14.7	2.57E-06
CI-994	93.1%	12.7	2.34E-07
Bromosporine	93.3%	17.8	2.30E-04
Scriptaid	93.5%	14.2	7.54E-06
UNC0224	93.9%	18.7	1.10E-03
PFI-1	94.0%	15.0	7.67E-05
3-Deazaneplanocin A (hydrochloride)	94.4%	15.2	2.25E-04
Pyroxamide	94.6%	13.8	1.60E-04
Sodium Butyrate	95.2%	15.7	2.07E-03
CBHA	95.2%	11.4	4.88E-05
Rucaparib (phosphate)	95.4%	13.8	8.70E-04
Pimelic Diphenylamide 106	95.6%	15.5	4.87E-03
Phthalazinone pyrazole	95.6%	17.7	1.32E-02
(-)-Neplanocin A	95.8%	14.6	4.35E-03
Tenovin-6	96.1%	14.7	7.80E-03
S-Adenosylhomocysteine	96.1%	14.7	8.55E-03
Splitomicin	96.1%	12.2	1.70E-03
GSK-J2 (sodium salt)	96.5%	10.9	1.71E-03
RVX-208	96.5%	11.2	2.51E-03
Methylstat (hydrate)	96.9%	13.3	1.96E-02
Decitabine	96.9%	12.1	3.28E-03
GSK126	96.9%	13.3	2.11E-02
Lomeguatrib	97.0%	11.5	9.54E-03
Ellagic Acid	97.0%	11.4	9.29E-03
IOX1	97.0%	10.7	6.67E-03
Etoposide	97.1%	13.3	2.89E-02
Tenovin-1	97.1%	10.3	6.56E-03
(R)-PFI-2 (hydrochloride)	97.3%	16.7	1.05E-01
Garcinol	97.4%	10.5	1.42E-02
UNC1999	97.4%	10.8	2.03E-02
DMOG	97.6%	12.8	6.15E-02
C646	97.6%	10.4	9.77E-03
Salermide	97.6%	10.5	2.88E-02
SGI-1027	97.7%	14.0	9.87E-02
Valproic Acid (sodium salt)	97.7%	9.9	2.46E-02
SGC0946	97.7%	12.2	6.90E-02
Chidamide	97.8%	11.6	6.86E-02
Tubacin	97.8%	11.9	7.07E-02
Mirin	97.9%	11.7	7.52E-02
EX-527	98.0%	10.7	6.81E-02
BRD73954	98.0%	9.1	3.51E-02
GSK4112	98.1%	10.4	7.37E-02
3,3'-Diindolylmethane	98.1%	11.0	9.53E-02
Butyrolactone 3	98.1%	9.7	6.72E-02
Sinefungin	98.2%	13.8	1.79E-01
5-Azacytidine	98.3%	9.3	7.55E-02
2',3',5'-triacetyl-5-Azacytidine	98.3%	8.8	6.60E-02
Tubastatin A (trifluoroacetate salt)	98.4%	8.7	7.07E-02
MI-2 (hydrochloride)	98.4%	10.4	1.27E-01
Zebularine	98.4%	11.8	1.92E-01
Sirtinol	98.4%	10.6	1.45E-01
(+)-Abscisic Acid	98.5%	11.1	1.85E-01
RSC-133	98.6%	9.4	1.65E-01
AK-7	98.7%	12.2	2.77E-01
Sodium 4-Phenylbutyrate	98.7%	11.7	2.60E-01
3-amino Benzamide	98.7%	10.6	2.21E-01
Anacardic Acid	98.7%	9.0	1.57E-01
HNHA	98.7%	9.3	1.77E-01
PCI 34051	98.8%	9.9	2.18E-01
S-(5'-Adenosyl)-L-methionine (tosylate)	98.8%	15.6	4.25E-01
trans-Resveratrol	98.9%	9.6	2.76E-01
I-CBP112 (hydrochloride)	99.0%	10.9	3.74E-01
Tubastatin A	99.0%	10.8	3.69E-01
Delphinidin (chloride)	99.1%	9.5	3.59E-01
RG-108	99.2%	8.0	3.52E-01
Octyl-a-ketoglutarate	99.3%	11.8	5.67E-01
BSI-201	99.4%	11.4	5.87E-01
GSK343	99.4%	10.8	5.73E-01
GSK-J1 (sodium salt)	99.4%	11.4	6.22E-01
SGC-CBP30	99.5%	10.0	5.77E-01
AGK2	99.5%	11.0	6.77E-01
CAY10591	99.6%	14.1	7.62E-01
2,4-Pyridinedicarboxylic Acid	99.6%	11.9	7.34E-01
CCG-100602	99.6%	11.1	7.35E-01
Piceatannol	99.6%	10.3	7.17E-01
a-Hydroxyglutaric Acid (sodium salt)	99.6%	10.2	7.23E-01
UNC0321 (trifluoroacetate salt)	99.7%	10.7	7.72E-01
Cl-Amidine (hydrochloride)	99.7%	8.4	7.47E-01
BML-210	99.8%	10.7	8.76E-01
N-Oxalylglycine	99.8%	10.2	8.81E-01
UNC1215	99.9%	12.5	9.19E-01
GSK-J4 (hydrochloride)	99.9%	9.6	9.00E-01
CAY10669	99.9%	11.7	9.25E-01
(-)-JQ1	99.9%	11.9	9.37E-01
MC 1568	99.9%	8.0	9.27E-01
GSK2801	100.0%	9.7	9.65E-01
JGB1741	100.1%	9.0	9.13E-01
1-Naphthoic Acid	100.3%	11.9	7.97E-01
CAY10683	100.3%	9.2	7.19E-01
Isoliquiritigenin	100.4%	10.8	7.06E-01
EPZ005687	100.4%	7.8	5.92E-01
5-Methyl-2'-deoxycytidine	100.5%	10.1	6.26E-01
WDR5-0103	100.5%	7.5	5.07E-01
Nicotinamide	100.5%	12.3	6.58E-01
RGFP966	100.5%	10.9	6.18E-01
F-Amidine (trifluoroacetate salt)	100.7%	9.1	4.81E-01
HPOB	100.7%	10.5	4.84E-01
Daminozide	100.8%	11.5	4.93E-01
2-PCPA (hydrochloride)	100.9%	9.1	3.14E-01
EPZ5676	101.0%	9.4	2.87E-01
PFI-3	101.2%	11.7	3.05E-01
Suramin (sodium salt)	101.3%	11.5	2.81E-01
2-hexyl-4-Pentynoic Acid	101.4%	10.9	1.99E-01
SIRT1/2 Inhibitor IV	101.6%	9.8	1.02E-01
GSK-LSD1 (hydrochloride)	102.1%	10.5	4.70E-02
AGK7	102.3%	10.8	3.96E-02
5-Methylcytidine	103.5%	11.3	2.48E-03

Supplementary Table S4. The most effective compound for each tumor sample. Table of the most effective compound for each of the 32 tumors screened. The most effective compound is determined as the compound that reduces the cell viability the most and has a p value less than 0.05. For the single sample (GAR) that did not have any compound which significantly reduced cell viability, the compound that reduced cell viability to the greatest extent is reported. Cell viability is reported as a percentage normalized to the untreated tumor control cohort and p values are calculated using the Mann-Whitney-U test (confidence level of 0.95).

Sample	Cell Viability	p Value	Compound
CH157*	28.6%	3.81E-03	CAY10398
GAR*	38.7%	1.17E-01	LAQ824
H23-3	40.4%	2.96E-03	UNC0631
IOMM Lee*	22.1%	8.15E-04	UNC0631
J10-6	46.6%	3.81E-03	Panobinostat
J11-2	53.0%	3.81E-03	JIB-04
J11-6	48.1%	3.81E-03	Panobinostat
J11-7	53.9%	3.81E-03	Panobinostat
J13-2	46.0%	3.72E-05	LAQ824
J15-4	82.2%	4.94E-05	JIB-04
J16-5	40.0%	3.81E-03	Panobinostat
J17-2	59.1%	3.81E-03	Panobinostat
J17-7	32.9%	3.81E-03	Panobinostat
J18-6	25.7%	3.70E-03	JIB-04
J18-7	47.8%	3.70E-03	Panobinostat
J19-2	28.5%	3.70E-03	Panobinostat
J19-3	35.7%	3.70E-03	JIB-04
J19-4	32.8%	3.70E-03	JIB-04
J19-5	37.4%	3.70E-03	Panobinostat
J19-6	30.5%	3.70E-03	JIB-04
J19-7	25.5%	3.70E-03	Panobinostat
J2-2	38.5%	3.53E-03	LAQ824
J21-4	50.0%	5.04E-05	Panobinostat
J21-5	36.0%	3.70E-03	Panobinostat
J21-7	52.7%	3.70E-03	Panobinostat
J4-1	88.8%	2.10E-02	OTX015
J4-4	24.0%	7.93E-04	BIX01294 (hydrochloride hydrate)
J7-6	28.7%	3.81E-03	HC Toxin
J8-3	51.1%	3.81E-03	OTX015
J8-4	21.4%	3.81E-03	LAQ824
J9-3	42.2%	3.68E-03	Panobinostat
JEN*	27.7%	1.81E-02	HC Toxin

Supplementary Table S5. Broadly effective epigenetic compounds separated by tumor grade. Epigenetic compounds that were broadly effective in (a) grade 1, (b) grade 2, and (c) grade 3 meningiomas. Broadly effective compounds for each grade are identified as compounds that reduce the average cell viability of the group by 80% or less and have a p value less than 0.05.

a) Grade 1 Broadly Effective Compounds

Compound	Avg. Cell Viability	Std. Deviation	p Value	Category
Panobinostat	51.9%	24.8	1.05E-30	Grade1
HC Toxin	59.3%	24.5	1.16E-25	Grade1
LAQ824	60.0%	24.0	1.89E-25	Grade1
Gemcitabine	73.9%	21.4	2.04E-18	Grade1
JIB-04	79.0%	27.8	1.10E-09	Grade1
CPI-203	79.6%	20.9	7.61E-14	Grade1

b) Grade 2 Broadly Effective Compounds

Compound	Avg. Cell Viability	Std. Deviation	p Value	Category
LAQ824	44.8%	16.8	1.67E-12	Grade2
Panobinostat	52.7%	19.1	3.04E-10	Grade2
HC Toxin	64.3%	24.5	1.69E-06	Grade2
Gemcitabine	65.6%	16.9	8.39E-09	Grade2
JIB-04	71.2%	31.4	4.50E-04	Grade2
SB939	77.0%	19.0	1.98E-05	Grade2
Apicidin	77.6%	22.4	1.81E-04	Grade2
Trichostatin A	78.6%	27.0	1.65E-03	Grade2
CAY10398	79.2%	24.8	1.01E-03	Grade2

c) Grade 3 Broadly Effective Compounds

Compound	Avg. Cell Viability	Std. Deviation	p Value	Category
UNC0631	22.1%	1.2	1.82E-35	Grade3
UNC0646	22.4%	2.9	1.39E-08	Grade3
MI-nc (hydrochloride)	24.2%	7.4	1.33E-04	Grade3
HC Toxin	27.6%	1.0	1.09E-27	Grade3
SB939	28.9%	4.2	3.50E-06	Grade3
CAY10603	30.2%	10.4	6.85E-04	Grade3
4-iodo-SAHA	31.5%	4.1	4.02E-06	Grade3
6-Thioguanine	31.7%	6.3	8.24E-05	Grade3
UNC0638	31.9%	19.6	5.86E-03	Grade3
Apicidin	32.1%	4.6	1.12E-05	Grade3
UNC0642	34.6%	25.4	1.40E-02	Grade3
LAQ824	39.1%	31.7	1.25E-02	Grade3
Panobinostat	46.6%	33.5	4.96E-02	Grade3
(-)-Neplanocin A	47.7%	11.4	2.35E-03	Grade3
CAY10398	50.1%	18.1	1.13E-02	Grade3
MS-275	50.3%	15.7	7.55E-03	Grade3
SAHA	53.3%	27.9	1.99E-02	Grade3
UNC0224	55.9%	24.1	1.47E-02	Grade3
CI-994	59.3%	22.8	3.72E-02	Grade3
3-Deazaneplanocin A (hydrochloride)	63.8%	12.4	9.14E-03	Grade3
3-Deazaneplanocin A	70.8%	15.9	1.43E-02	Grade3

Supplementary Table S6. Comparison of compound effectiveness across tumor grade. p values for the comparison of cell viabilities for broadly effective compounds across grade 1, 2, and 3 meningiomas. Comparisons were conducted with an ANOVA followed by a Tukey HSD post-hoc test (confidence level of 0.95) by comparing compound sensitivity across tumor grade.

	p Value		
**Compound**	**Grade2-Grade1**	**Grade3-Grade1**	**Grade3-Grade2**
(-)-Neplanocin A	3.10E-01	1.11E-13	3.54E-11
4-iodo-SAHA	3.09E-02	7.67E-12	2.29E-08
6-Thioguanine	9.81E-01	5.21E-07	3.40E-06
Apicidin	5.46E-01	1.89E-05	3.61E-04
CAY10398	3.02E-01	1.15E-03	1.82E-02
CAY10603	5.11E-02	1.24E-10	1.67E-07
CPI-203	1.86E-03	7.56E-01	6.42E-01
Gemcitabine	2.67E-01	8.76E-02	3.96E-01
HC Toxin	6.74E-01	7.15E-02	4.13E-02
JIB-04	5.15E-01	8.84E-01	9.94E-01
LAQ824	2.28E-02	1.28E-01	8.75E-01
MI-nc (hydrochloride)	4.12E-01	1.75E-10	2.44E-08
Panobinostat	9.90E-01	9.05E-01	8.89E-01
SB939	5.25E-01	1.18E-05	2.57E-04
Trichostatin A	5.43E-01	6.47E-03	4.11E-02
UNC0631	6.29E-01	2.43E-05	1.47E-05
UNC0638	6.52E-01	4.58E-05	5.78E-04
UNC0642	3.74E-01	2.04E-08	1.80E-06
UNC0646	4.99E-01	1.70E-06	5.38E-05

Supplementary Table S7. Broadly effective epigenetic compounds separated by primary and recurrent tumors. Epigenetic compounds that were broadly effective in (a) primary and (b) recurrent meningiomas. Broadly effective compounds for each grade are identified as compounds that reduce the average cell viability of the group to 80% or less and have a p value less than 0.05.

a) Primary Tumor Broadly Effective Compounds

Compound	Avg. Cell Viability	Std. Deviation	p Value	Category
Panobinostat	52.1%	24.321	2.34E-36	Primary
LAQ824	56.2%	23.206	6.62E-34	Primary
HC Toxin	59.2%	24.333	2.88E-30	Primary
Gemcitabine	71.5%	20.976	2.11E-24	Primary
JIB-04	78.2%	28.116	1.09E-11	Primary

b) Recurrent Tumor Broadly Effective Compounds

Compound	Avg. Cell Viability	Std. Deviation	p Value	Category
Panobinostat	49.0%	20.404	2.28E-05	Recurrent
LAQ824	55.0%	31.534	7.91E-04	Recurrent
HC Toxin	60.9%	30.229	4.64E-03	Recurrent
Apicidin	61.9%	28.895	2.41E-03	Recurrent
SB939	62.7%	29.897	3.37E-03	Recurrent
UNC0631	63.9%	36.444	1.21E-02	Recurrent
MI-nc (hydrochloride)	65.8%	36.403	1.57E-02	Recurrent
CAY10603	66.3%	33.007	1.04E-02	Recurrent
UNC0646	67.0%	39.477	2.68E-02	Recurrent
6-Thioguanine	67.1%	32.388	1.06E-02	Recurrent
4-iodo-SAHA	67.2%	31.897	1.00E-02	Recurrent
JIB-04	69.2%	35.850	1.74E-02	Recurrent
UNC0638	69.5%	34.401	2.06E-02	Recurrent
Gemcitabine	70.2%	34.427	2.31E-02	Recurrent
Trichostatin A	70.5%	29.663	1.18E-02	Recurrent
BIX01294 (hydrochloride hydrate)	74.3%	35.547	4.83E-02	Recurrent
CAY10398	76.6%	25.232	1.67E-02	Recurrent
UNC0224	77.3%	27.308	2.02E-02	Recurrent
(-)-Neplanocin A	77.4%	27.013	2.65E-02	Recurrent
M 344	78.9%	29.500	4.98E-02	Recurrent
CI-994	80.0%	22.717	2.12E-02	Recurrent

Supplementary Table S8. Comparison of compound effectiveness across primary and recurrent tumors. p values for the comparison of cell viabilities for broadly effective compounds across primary and recurrent meningiomas. Comparisons were conducted with the Student's t test (confidence level of 0.95) by comparing compound sensitivity between primary and recurrent tumors.

	p Value
Compound	Primary-Recurrent
4-iodo-SAHA	4.93E-02
6-Thioguanine	1.29E-01
Apicidin	5.91E-02
CAY10603	6.22E-02
Gemcitabine	9.08E-01
HC Toxin	8.79E-01
JIB-04	4.40E-01
LAQ824	9.02E-01
MI-nc (hydrochloride)	8.98E-02
Panobinostat	6.56E-01
SB939	8.42E-02
Trichostatin A	2.29E-01
UNC0631	1.31E-01
UNC0638	1.27E-01
UNC0646	1.88E-01

Supplementary Table S9. Broadly effective epigenetic compounds separated by tumor radiation history. Epigenetic compounds that were broadly effective in (a) tumors with no prior history of radiation, (b) tumors with a prior history of radiation and (c) tumors with a non-specified history of radiation. Broadly effective compounds for each grade are identified as compounds that reduce the average cell viability of the group to 80% or less and have a p value less than 0.05.

a) Tumors with No History of Radiation Broadly Effective Compounds

Compound	Avg. Cell Viability	Std. Deviation	p Value	Category
Panobinostat	52.5%	23.1	7.82E-35	No Radiation
LAQ824	59.0%	23.0	6.51E-30	No Radiation
HC Toxin	62.7%	24.6	9.51E-26	No Radiation
Gemcitabine	73.8%	18.7	1.38E-23	No Radiation
JIB-04	77.7%	28.6	4.57E-11	No Radiation

b) Tumors with a Prior History of Radiation Broadly Effective Compounds

Compound	Avg. Cell Viability	Std. Deviation	p Value	Category
JIB-04	25.7%	9.6	4.29E-03	Prior Radiation
Panobinostat	31.8%	1.4	2.81E-16	Prior Radiation
LAQ824	35.8%	8.5	4.18E-03	Prior Radiation
HC Toxin	42.2%	8.4	5.22E-03	Prior Radiation
CPI-203	43.5%	1.3	7.50E-18	Prior Radiation
(+)-JQ1	48.3%	17.3	3.43E-02	Prior Radiation
Gemcitabine	53.2%	2.5	3.08E-06	Prior Radiation
Lestaurtinib	55.0%	4.5	8.91E-04	Prior Radiation
Apicidin	60.2%	10.9	2.17E-02	Prior Radiation
I-BET762	64.0%	18.3	4.46E-03	Prior Radiation
Bromosporine	68.6%	11.8	4.15E-02	Prior Radiation
6-Thioguanine	71.1%	4.6	3.23E-03	Prior Radiation
BSI-201	73.0%	7.2	1.77E-02	Prior Radiation
Phthalazinone pyrazole	73.0%	5.8	9.51E-03	Prior Radiation
3-Deazaneplanocin A (hydrochloride)	75.4%	6.9	1.92E-02	Prior Radiation
2,4-Pyridinedicarboxylic Acid	76.7%	8.4	3.53E-02	Prior Radiation
UNC0631	77.6%	4.5	5.89E-03	Prior Radiation
(-)-JQ1	78.9%	8.7	4.62E-02	Prior Radiation
Trichostatin A	79.4%	4.8	8.99E-03	Prior Radiation

c) Tumors with a Non-Specified History of Radiation Broadly Effective Compounds

Compound	Avg. Cell Viability	Std. Deviation	p Value	Category
HC Toxin	35.7%	9.2	3.07E-13	Not Specified
SB939	39.0%	11.3	6.21E-14	Not Specified
UNC0646	40.5%	27.1	1.22E-06	Not Specified
LAQ824	41.5%	26.0	8.74E-07	Not Specified
6-Thioguanine	45.4%	21.6	5.88E-08	Not Specified
MI-nc (hydrochloride)	50.0%	25.7	2.00E-06	Not Specified
CAY10398	50.6%	23.6	1.92E-06	Not Specified
Apicidin	51.1%	21.3	5.81E-07	Not Specified
Panobinostat	52.2%	30.1	2.20E-05	Not Specified
SAHA	53.9%	22.3	2.18E-06	Not Specified
CAY10603	55.7%	28.4	2.70E-05	Not Specified
Trichostatin A	56.9%	24.8	7.82E-06	Not Specified
BIX01294 (hydrochloride hydrate)	57.4%	33.4	3.58E-04	Not Specified
UNC0642	59.3%	30.3	1.29E-04	Not Specified
Gemcitabine	59.5%	37.7	1.47E-03	Not Specified
UNC0638	59.7%	35.6	9.72E-04	Not Specified
M 344	62.2%	24.4	5.35E-05	Not Specified
UNC0631	63.1%	38.2	2.19E-03	Not Specified
Oxamflatin	64.1%	21.0	1.86E-05	Not Specified
MS-275	67.2%	23.1	6.99E-05	Not Specified
4-iodo-SAHA	71.8%	29.2	3.18E-03	Not Specified
3-Deazaneplanocin A	78.6%	13.6	1.85E-05	Not Specified
CI-994	78.6%	20.1	2.38E-03	Not Specified
UNC0224	78.7%	24.0	4.14E-03	Not Specified
ITF 2357	79.2%	26.9	1.68E-02	Not Specified
(-)-Neplanocin A	79.3%	23.4	5.92E-03	Not Specified

Supplementary Table S10. Broadly effective epigenetic compounds separated by patient gender. Epigenetic compounds that were broadly effective in tumors originating from (a) female and (b) male patients. Broadly effective compounds for each grade are identified as compounds that reduce the average cell viability of the group to 80% or less and have a p value less than 0.05.

a) Female-Derived Patient Tumors Broadly Effective Compounds

Compound	Avg. Cell Viability	Std. Deviation	p Value	Category
Panobinostat	53.1%	23.7	4.10E-31	Female
LAQ824	58.4%	23.9	3.75E-27	Female
HC Toxin	62.8%	24.8	1.10E-23	Female
Gemcitabine	73.5%	19.0	7.98E-22	Female
JIB-04	74.5%	30.2	1.69E-11	Female

b) Male-Derived Patient Tumors Broadly Effective Compounds

Compound	Avg. Cell Viability	Std. Deviation	p Value	Category
HC Toxin	45.3%	19.3	1.14E-11	Male
Panobinostat	47.6%	24.8	9.62E-11	Male
LAQ824	48.1%	23.2	7.08E-11	Male
SB939	55.2%	25.1	6.71E-09	Male
6-Thioguanine	61.2%	25.0	4.29E-08	Male
UNC0646	63.9%	36.6	7.28E-05	Male
Gemcitabine	63.9%	30.8	7.70E-06	Male
Apicidin	64.9%	24.6	3.53E-07	Male
BIX01294 (hydrochloride hydrate)	66.0%	34.1	6.40E-05	Male
UNC0631	66.9%	35.1	8.78E-05	Male
CAY10398	69.8%	25.6	6.61E-06	Male
Trichostatin A	70.6%	24.4	3.04E-06	Male
UNC0638	70.9%	37.5	9.62E-04	Male
MI-nc (hydrochloride)	71.0%	31.5	1.18E-04	Male
CAY10603	72.5%	28.8	7.67E-05	Male
SAHA	72.5%	28.6	3.68E-05	Male
M 344	74.9%	22.2	1.30E-05	Male
CPI-203	75.9%	19.9	3.08E-06	Male
UNC0642	76.2%	33.2	1.95E-03	Male
Oxamflatin	78.1%	20.4	2.55E-05	Male
4-iodo-SAHA	79.7%	24.3	4.75E-04	Male
MS-275	79.9%	18.6	1.69E-05	Male

Supplementary Table S11. Comparison of compound effectiveness between female and male-derived patient tumors. p values for the comparison of cell viabilities for broadly effective compounds between meningiomas originated from female and male patients. Comparisons were conducted with the Student's t test (confidence level of 0.95) by comparing compound sensitivity between tumors derived from female and male patients.

Compound	p Value Male-Female
6-Thioguanine	9.56E-06
Apicidin	1.25E-03
BIX01294 (hydrochloride hydrate)	1.05E-03
CAY10398	3.49E-03
CAY10603	4.56E-03
Gemcitabine	1.60E-01
HC Toxin	1.19E-03
JIB-04	2.79E-02
LAQ824	6.40E-02
MI-nc (hydrochloride)	6.38E-03
Panobinostat	3.34E-01
SB939	3.20E-06
Trichostatin A	1.06E-02
UNC0631	1.57E-02
UNC0638	1.98E-02
UNC0646	3.48E-03

Supplementary Table S12. Compounds used in this study. The 139-compound library utilized in this study, including the primary targets for each of these compounds.

Compound	Target
(-)-JQ1	BET proteins, no specific affinity as +-JQ1 does.
(-)-Neplanocin A	SAH hydrolase, Ki 8.39nM.
(+)-Abscisic Acid	Ubiquitous signalling molecule, in humans is known for anti-inflamatory and cellular metabolic reprogramming.
(+)-JQ1	BET proteins, notably BRD4.
(R)-PFI-2 (hydrochloride)	SET7/9, IC50 2nM.
1-Naphthoic Acid	Precursor molecule.
2-hexyl-4-Pentynoic Acid	HDACs.
2-PCPA (hydrochloride)	LSD1, IC50 20.7uM.
2,4-Pyridinedicarboxylic Acid	Chelator of Zinc and involved in JMJ protein pathways.
2',3',5'-triacetyl-5-Azacytidine	Inhibitor of DNA methyltransferase.
3-amino Benzamide	Poly(ADP-ribose) polymerases, Ki = 1.8μM.
3-Deazaneplanocin A	S-adensoyl-L-homocystine hydrolase inhibitor.
3-Deazaneplanocin A (hydrochloride)	Inhibits trimethylation of lysine on histone H3.
3,3'-Diindolylmethane	Nrf2 inhibitor.
4-iodo-SAHA	HDAC1, >60% inhibition at 1μM.
5-Azacytidine	DNA methyltransferase inhibitor.
5-Methyl-2'-deoxycytidine	Incorporates into single strand DNA.
5-Methylcytidine	Incorporates into RNA, 1 to 2 per 1,000 residues.
6-Thioguanine	DNA methyltransferase inhibitor.
a-Hydroxyglutaric Acid (sodium salt)	L-2-dehydrogenases inhibitor.
AGK2	SIRT2 inhibitor.
AGK7	SIRT2 inhibitor, IC50 3.5uM.
AK-7	SIRT2 inhibitor, IC50 15.5uM.
Anacardic Acid	Inhibitor of HATs p300 and p300/CREB.
Apicidin	HDACs, IC50 ~ 0.7 nM.
BIX01294 (hydrochloride hydrate)	G9a HMTase, IC50 1.7uM.
BML-210	HDAC, IC50 30uM.
BRD73954	HDAC6, IC50 36nM.
Bromosporine	Non-specific brodomain inhibitor.
BSI-201	PARP1 inhibitor.
Butyrolactone 3	Histone acetyltransferase Gcn5, IC50 100uM.
C646	Histone acetyltransferase p300, IC50 = 1.6μM, Ki = 400 nM.
CAY10398	HDAC1, IC50 12uM.
CAY10591	SIRT1
CAY10603	HDAC6, other HDACs to lower extents.
CAY10669	Histone acetyltransferase PCAF.
CAY10683	HDAC2, IC50 0.119nM
CBHA	HDACs
CCG-100602	Inhibitor Rho pathway-mediated signaling
Chidamide	Increases histon H3 acetyltion levels
CI-994	HDACs 1,2,3,8
Cl-Amidine (hydrochloride)	Protein arginine deiminases (PAD1, PAD3, PAD4)
coumarin-SAHA	Class I and II HDACs. Coumarin is a flourescent tag for SAHA
CPI-203	BRD4
CPTH2 (hydrochloride)	Inhibitor of HAT activity of Gcn5
CUDC-101	HER1, HER2, IC50 2.4nM, 16.4nM
Daminozide	2OGs: KDM2A, PHF8, KDM7A
Decitabine	DNA methyltransferases
Delphinidin (chloride)	ER-alpha, ultimately EGFR
DMOG	HIF-1alpha
Ellagic Acid	Blocks methylation of argine 12 on histone 3 through CARM1
EPZ005687	EZH2
EPZ5676	DOT1L histone methytrasferase, Ki 80pM
Etoposide	Nulear receptor coactivator
EX-527	SIRT1, IC50 98nM
F-Amidine (trifluoroacetate salt)	PAD1, PAD4
Garcinol	Histone acetyltransferases (HAT) p300, IC50 ~ 7μM
Gemcitabine	DNA damage inducible protein 45a
GSK-J1 (sodium salt)	Inhibitor of H3K27 histone demethylases: JMJD3, UTX
GSK-J2 (sodium salt)	Negative control for above GSK-J1, poorly inhibits JMJD3
GSK-J4 (hydrochloride)	Inhibits JMJD3, less effective than GSK-J1, but more permeable to cells.
GSK-LSD1 (hydrochloride)	LSD1, IC50 16nM
GSK126	EZH2 methyltransferase, IC50 9.9nM
GSK2801	BAZ2A
GSK343	EZH2, ultimtely acts on H3K27.
GSK4112	REV-ERB-alpha, ultimately impacting circadian target gene bmal1.
HC Toxin	HDACs
HNHA	HDACs
HPOB	HDAC6, IC50 56nM
I-BET151	BRD2, BRD3, BRD4
I-BET762	BET proteins, including BRD2, BRD3, BRD4.
I-CBP112 (hydrochloride)	CBP
IOX1	2OG oxygenases: PHF8, PHD2, FIH
Isoliquiritigenin	Estrogen receptor alpha and beta antagonist
ITF 2357	class I and II HDACs
JGB1741	SIRT1, IC50 15uM
JIB-04	Jumonji histone demethylases
LAQ824	HDAC, IC50 30uM
Lestaurtinib	JAK2, IC50 1nM
Lomeguatrib	MGMT
M 344	HDACS, esp HDAC6, then HDAC1
MC 1568	Class IIa HDAC
Methylstat (hydrate)	JMJD2A, JMJD2C, JMJD2C, JMJD2E, JMJD3
MI-2 (hydrochloride)	Binds to menin, blocking menin-MLL fusion protein interaction
MI-nc (hydrochloride)	Weaker version of above MI-2, worse blocker of menin-MLL
Mirin	MRN, thus inhibiting MRN dependent phopsphorylation of histone H2AX and prevents ATM activation
MS-275	HDAC1, HDAC3
N-Oxalylglycine	JMJD2A, HMHD2C, JMJD2E, IC50 250, 500, 24 uM
Nicotinamide	SIRT1
Octyl-a-ketoglutarate	Stimulates PHD activity, increasing HIF-1alpha turnover
OTX015	BRD2, BRD3, BRD4, IC50 levels 10-19nM
Oxamflatin	HDACs
Panobinostat	Broad-spectrum HDAC inhibitor
PCI 34051	HDAC8, IC50 = 0.01 ~μM
PFI-1	BRD2, BRD4, other brodomain things
PFI-3	SMARCA4
Phthalazinone pyrazole	Aurora A Kinase, IC50 31nM
Piceatannol	IkB-alpha
Pimelic Diphenylamide 106	Class I HDACs
Pyroxamide	HDAC1
RG-108	DNA methyltransferase, IC50 115nM
RGFP966	HDAC3, IC50 .08uM
RSC-133	DNA methyltransferase
Rucaparib (phosphate)	PARP1
RVX-208	BD2, IC50 0.04-0.28uM
S-(5'-Adenosyl)-L-methionine (tosylate	Ubiquitous methyl donor
S-Adenosylhomocysteine	Controls levels of AdoHcy, which is an inhibitor of some methyltransferases.
SAHA	HDAC I, 50-200nm
Salermide	SIRT1
SB939	HDAC isozymes, Ki = 16-26 nM
Scriptaid	HDAC
SGC-CBP30	CREPBP, IC50 21-69nM
SGC0946	DOT1L, IC50 0.3nM
SGI-1027	DNMT1, DNMT3A, DNMT3B
Sinefungin	SET domain containing methyltransferases.
SIRT1/2 Inhibitor IV	SIRT1, IC50 56uM
Sirtinol	Sirtuin NAD+-dependent deacetylases, IC50 ~ 60μM
Sodium 4-Phenylbutyrate	Weak inhibitor of HDACs
Sodium Butyrate	HDACs
Splitomicin	Sir2p
Suramin (sodium salt)	Ryanodine receptor-1
Tenovin-1	SIRT1
Tenovin-6	SIRT2, IC50 10uM
trans-Resveratrol	COX-1
Trichostatin A	HDAC1, 70nM
Tubacin	HDAC6, IC50 4nM
Tubastatin A	HDAC6, IC50 15nM
Tubastatin A (trifluoroacetate salt)	HDAC6, IC50 ~ 15 nM
UNC0224	G9a HMTase, IC50 15nM
UNC0321 (trifluoroacetate salt)	G9a HMTase
UNC0631	G9a
UNC0638	G9a HMTase inhibitor
UNC0642	G9a, GLP, ultimately H3K9
UNC0646	G9a/GLP
UNC1215	Kme reading function of L3MBTL3, IC50 40nM
UNC1999	EZH2, IC50 2nM
Valproic Acid (sodium salt)	Class I HDACs
WDR5-0103	WDR5
Zebularine	DNMTs
